# A dynamic micro-macro-economic model to assess water charging policies

**DOI:** 10.1016/j.jclepro.2026.147802

**Published:** 2026-03-01

**Authors:** Francesco Sapino, Ramiro Parrado, C. Dionisio Pérez-Blanco

**Affiliations:** a IMDEA Water Institute, Alcalá de Henares, Spain; b CMCC Foundation - Euro-Mediterranean Center on Climate Change, Venice, Italy; c RFF-CMCC European Institute on Economics and the Environment, Venice, Italy; d Università Ca’Foscari, Venice, Italy

**Keywords:** Agricultural economics, CGE, Mathematical programming, Water management

## Abstract

This paper presents an integrated and dynamic coupling of micro- and macro-economic models for water resources management. As compared to conventional static micro-macro-economic coupling designs, the dynamic coupling produces time-variant assessments of endogenous (e.g., dynamics and impacts of capital accumulation) and exogenous variables (e.g., impacts of policy shifts or market trends over time). Methods are illustrated with an application to the Castile and León Region in Spain, where we: 1) assess the performance of two hypothetical water charging policies (one-time charge adjustment v. Incremental charges), and 2) compare the simulation results obtained with the proposed dynamic coupling to those obtained using two conventional static models. A key finding is that simulations under the dynamic coupling attribute lower effectiveness to water charges in reducing water use over time: 47% and −24% (dynamic coupling) water use change at the beginning and end of the simulation period vs. −46% (static coupling). Additionally, an incremental water charge has a greater economic impact than a one-time adjustment, resulting in a peak profit reduction of −8.9% compared to −5.9% under the one-time charge. Our results highlight the relevance of accounting for dynamic micro-macro-economic dynamic feedback in water policy design.

## 1 Introduction

Technological development and investments in water works have allowed societies to harness the potential of water resources to grow crops, generate energy, and manufacture commodities beyond the needs of direct water users (Hanemann, 2006). Growing surpluses have driven market exchanges at a local, regional, and international level, interconnecting water bodies and direct (such as farmers) and indirect water users (such as food industry). Increased interconnectedness has also grown economic exposure, thus amplifying the economy-wide impacts of water scarcity and droughts, whose incidence is increasing under climate change (Fitch Wire, 2022; Haqiqi et al., 2023; Peiris et al., 2022; UNDRR, 2021). In some arid regions, water scarcity and droughts have already reduced GDP by up to 2.5%, with projections estimating up to 6% reduction by 2030 if current water use trends persist (UNESCO, 2020; World Bank, 2021).

Understanding the local and economy-wide repercussions of water scarcity and droughts, as well as those of the water policies designed to prevent or alleviate them, calls for models that can reproduce key processes and interactions at the micro- and macro-economic systems level. A common feature of the micro-macro-economic coupled models developed to address this need is that they adopt a static design. Static micro-macro-economic coupled models are useful to assess the economic impacts of water scarcity and/or policies under stationary conditions where systems remain stable over time (Parrado et al., 2019). On the other hand, consideration of the time dimension is necessary to model economic systems under non-stationary conditions, including due to climate change, policy shocks, or capital accumulation processes that change statistical properties of key variables such as prices or factor productivity (Ferrari et al., 2022).

This paper develops an innovative two-way *dynamic* modular coupling framework for micro- and macro-economic models that can assess the changing economic impacts of water scarcity and/or policies *over time*. The microeconomic module is populated with a Positive Multi-Attribute Utility Programming (PMAUP) model that works at the level of water users (irrigators), while the macroeconomic one is populated with a Computable General Equilibrium (CGE) model that works at a regional (NUTS2) and sectorial level. Micro- and macro-economic systems iteratively and dynamically exchange information on land uses (micro-to macro-economic), crop prices (macro-to micro-economic) and yield changes (macro-to micro-economic). Moreover, compared to previous micro-macro-economic coupled models (Parrado et al., 2020), our approach offers a threefold increase in spatial granularity, enabling a more detailed assessment of the microeconomic impacts of water policies while preserving hydrological boundaries and integrity. Methods are illustrated with an application to the Castille and Leon region, Spain, where we: 1) assess the performance of two hypothetical water charging policies (one-time charge adjustment v. Incremental charges), and 2) compare the simulation results obtained with the dynamic micro-macro-economic coupling to those obtained using two conventional static models: a micro-macro-economic coupled model and a standalone microeconomic model.

The paper is structured as follows. Section 2 provides a literature review of micro–macro-economic coupled models for water scarcity and reallocation assessment; Section 3 presents the case study used to illustrate our dynamic coupling framework; Section 4 presents the methodology; Section 5 presents and discusses the results; and Section 6 provides concluding remarks and suggestions for future research.

## 2 Literature review of micro-macro-economic coupled models

Traditionally, the assessment of the socioeconomic impacts of water scarcity and reallocations has relied on microeconomic models. Microeconomic models aim at understanding how individual users, notably farmers, respond to changes in water availability, prices, and policies, and how these responses affect resource use and income distribution. Please note that we did not include a detailed review of microeconomic models here because the literature is extensive and has already been comprehensively surveyed elsewhere—for example, in Soriano et al. (2025), which reviews microeconomic models for water (re)allocation. Variables such as yields per hectare and commodity and input prices are assumed to be exogenous, while endogenous variables include land and water allocation. However, users’ responses influence quantities and prices in their own markets (e.g., agricultural commodities), as well as those of other interlinked markets (e.g., food retail), and ignoring these interlinkages risks providing partial and potentially misleading information on the consequences of water scarcity and water reallocation policies (Loch et al., 2020).

One way to capture the broader economic impacts of water scarcity and reallocations is through standalone macroeconomic models. Macroeconomic models applied to water scarcity and reallocations include Input-Output (IO) models (González, 2011; Llop, 2008; Oosterhaven and Bouwmeester, 2016), partial equilibrium macroeconomic models (Rosegrant et al., 1995), and CGE models (Berrittella et al., 2007; Calzadilla et al., 2011; Liu et al., 2014). These models are useful for exploring economy-wide linkages, but they rely on coarse representative agents (e.g., a single “agricultural sector” per region, nation or even a group of nations) that reduce granularity and detail, potentially omitting relevant factors driving user behavior (constraints, technologies, attributes) (González, 2011) and making unrealistic assumptions on water dynamics such as free reallocations within the representative agent's space, which may not be feasible due to the bulkiness of water (moving water upstream or between basins can be costly, or even unfeasible) (Taheripour et al., 2013).

To overcome these limitations, researchers have developed integrated micro–macro-economic coupling frameworks that combine the behavioral detail and spatial granularity of microeconomic models with the broader scope and sectoral interlinkages of macroeconomic models. Two main approaches exist holistic or modular. In holistic approaches, micro- and macro-economic elements are fully integrated into a single model that is solved at once, requiring the simplification of some elements of the original models—typically microeconomic models. As a result, holistic models tend to face similar limitations to those of standalone macroeconomic models, including low granularity and behavioral detail (Calzadilla et al., 2011; Darwin et al., 1995) and free circulation of water resources across disconnected catchments (Hertel and Liu, 2016; Taheripour et al., 2013). In modular approaches, both micro- and macro-economic models are run independently in modules, which are subsequently interconnected using protocols. The use of a modular approach provides higher spatial granularity, behavioral detail, and flexibility as compared to holistic models (Britz et al., 2021), but increases computational costs and can lead to inconsistencies unless protocols are adequately designed (Essenfelder et al., 2018). Modularity is the predominant approach in the literature on integrated micro-macro-economic modeling of water scarcity and reallocations, with multiple coupling methods existing (Parrado et al., 2019).

Among modular approaches, one-way couplings are the most common. In these models, the outputs of the microeconomic model are used as an input to run macroeconomic simulations and assess economy-wide repercussions. Pérez-Blanco et al. (2017) implemented such coupling via a one-way protocol between a microeconomic PMAUP model and a macroeconomic I-O model, while Pérez-Blanco and Standardi (2019) applied a similar approach to a CGE model. Haqiqi et al. (2022) coupled a water balance model with SIMPLE-G-Global (an agroeconomic global model) to capture the global economic response to sustainable groundwater use. Similarly, MAgPIE incorporates various aspects of the biophysical system and simulates regional land allocation minimizing the “total cost of production for a given amount of regional food and bioenergy demand”, considering grid-based potential crop yields, land, and water from the dynamic global vegetation model LPJmL (Dietrich et al., 2022). A limitation of modular sequential applications is that they focus on transferring data from micro-to macro-economic models and disregard the impacts that production and price changes at a macroeconomic level can have in turn on the decisions of microeconomic agents, thus neglecting potentially relevant two-way feedback emerging across space and over time (the modular sequential models above are static).

Iterative or two-way coupling approaches address this limitation by sequentially exchanging data between the micro and macro modules until convergence is reached (both models reach a stable equilibrium). CAPRI (Britz, 2008) is one such model, linking a regional partial equilibrium macroeconomic model with a microeconomic Positive Mathematical Programming (PMP) model to analyze EU agricultural policy impacts. In an alternative application, Britz and Hertel (2011) linked CAPRI's microeconomic PMP model with a CGE model, calibrating price elasticities and integrating results without further iteration. Noteworthy, these CAPRI-based couplings do not explicitly model water, being used to analyze agricultural policies and shocks other than water scarcity and reallocations. CAPRI Water (Blanco et al., 2018) addressed this gap by adding a water module capable of explicitly modeling irrigation and rainfed agriculture. The main limitation of CAPRI-based models is that the EU-wide spatial coverage comes at the expense of reduced spatial granularity (NUTS 2 level, including an entire region or group of regions), which may be inadequate for assessing water reallocations and policy. More recently, Parrado et al. (2020, 2019) adopted a two-way coupling protocol between a microeconomic PMAUP model and a CGE model that exchange information on land use (micro-to macro-economic) and commodity prices (macro-to micro-economic) using a static approach that run simulations iteratively until convergence is achieved. Pérez-Blanco et al. (2022) and Valle-García et al. (2025) further extended this framework by incorporating a water system module interconnected with the microeconomic model—PMAUP in Pérez-Blanco et al. (2022) and PMP in Valle-García et al. (2025)—thereby linking it indirectly to the macroeconomic module through two-way feedbacks on water availability and use. Table 1 summarizes the main contributions of the different models described above.

**Table 1:** Integrated Micro- and Macro-Economic Modelling Frameworks for Water Scarcity and Allocation Policy. Microeconomic models alone were not included in this table, for a review of these models for water (re)allocation, see Soriano et al. (2025).

Authors	Key system modeled	Model	Coupling setup	Spatial granularity	Behavioral detail
Rosegrant et al. (1995)	Macroeconomic	IMPACT (PE)	No coupling	Medium (National)	Low
Darwin et al. (1995)	Macroeconomic	CGE	No coupling	Low (Global: 8 regions)	Low
Berrittella et al. (2007)	Macroeconomic	CGE	No coupling	Low (Global: 16 regions)	Low
Llop (2008)	Macroeconomic	I-O	No coupling	Low (National)	Low
Britz and Hertel (2011)	Micro & Macro-economic	PMP-CGE	One-way	Medium in macro (global regions by AEZ); Medium in mciro (EU regions)	Medium
González (2011)	Macroeconomic	I-O	No coupling	Medium (Regional)	Low
Calzadilla et al. (2011)	Macroeconomic	CGE	No coupling	Low (Global: 16 regions)	Low
Taheripour et al. (2013)	Macroeconomic	CGE	No coupling	Low (Global: 16 regions, but aggregated from 126 river basins)	Low
Liu et al. (2014)	Macroeconomic	CGE	No coupling	Low (Global: 16 regions, but aggregated from 126 river basins)	Low
Oosterhaven and Bouwmeester (2016)	Macroeconomic	I-O	No coupling	Medium (Regional)	Low
Pérez-Blanco et al. (2017)	Micro- & Macroeconomic	PMAUP & I-O	One-way	High in micro (agricultural region), medium in macro module (20 NUTS2 regions for Italy and rest of EU)	High
Blanco et al. (2018)	Micro & Macro-economic	CAPRI (PMP-PE)	Iterative two-way	Medium (NUTS-2 supply regions with rain-fed/irrigated split; 77 countries/40 trade blocks in market module)	Medium
Pérez-Blanco and Standardi (2019)	Micro & Macro-economic	PMAUP & CGE	One-way	High in micro module (irrigation districts); high in macro module (17 NUTS2 regions for Spain + supra-national regions)	High
Parrado et al. (2019)	Micro- & Macro-economic	PMAUP & CGE	Iterative two-way	High in micro (agricultural distrcit), high in macro module (17 NUTS2 regions for Spain + supra-national regions)	High
Haqiqi et al. (2022)	Hydrologic, agronomic, and macroeconomic	WBM-SIMPLE-G	One-way	High (5 arc-min grid cells for agro-economic model; 30 arc-min for hydrologic model), low in macro (17 global regions)	Low
Dietrich et al. (2022)	Hydrologic, agronomic, and macroeconomic	MAgPIE-LPJmL	Iterative two-way (decadal time steps)	High in agro-hydro processes (0.5° grid), low in macro (10 global economic regions)	Low
Pérez-Blanco et al. (2022)	Hydrologic, Micro & Macro-economic	SWAT-PMAUP-CGE	Iterative two-way	High in micro (agricultural distrcit), high in macro module (17 NUTS2 regions for Spain + supra-national regions)	High
Valle-García et al. (2025)	Hydrologic, Micro & Macro-economic	Hydro-economic (PMP)-CGE	Iterative two-way	High in micro (irrigation district), high in macro module (17 NUTS2 regions for Spain + supra-national regions)	High

Legend: IMAPCT (International Model for Policy Analysis of Agricultural Commodities and Trade), PE (Partial Equilibrium), WBM (Water Balance Model), SWAT (Soil and Water Assesment Tool).

Summing up, the literature on micro- and macro-economic assessments of water scarcity and reallocations ignores time dynamics and often works at coarse scales that limit behavioral detail (e.g., normative objective functions) and overlook hydrological integrity (e.g., allowing water reallocations between disconnected basins). This paper proposes a methodological framework to address these limitations.

## 3 Case study area

**Fig. 1:**
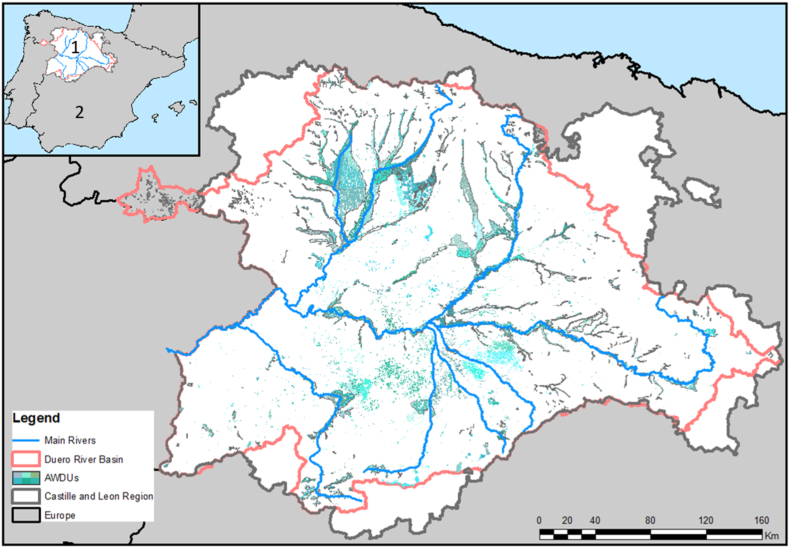
Case study area. AWDUs are located within the Castille and Leon region (1) and the Duero River basin, in Spain (2).

Methods are illustrated with an application to Spain's Castile and León Region (Fig. 1), which has an area of 94,226 km^2^ (18.6% of Spain territory). Despite its vast size, it has a relatively small population of 2,519,875 inhabitants, making up 5.39% of Spain's total population. Agriculture plays a substantial role in the region, contributing 5.26% of its GDP and employing 6.2% of its workforce, significantly exceeding national averages (2.8% and 4%, respectively) (INE, 2024, 2023). Key crops include wheat, barley, rye, oats, legumes (e.g., carob and chickpeas), sunflower, and vineyard (Table 2).

**Table 2:** Crops (in the PMAUP model) and crops category (in the CGE model) and their irrigated and rainfed area in 2017.

Crop categories (CGE model)	Individual crops (PMAUP model)	Irrigated area (ha)	Rainfed area (ha)
Wheat	Wheat	40770	688912

Other cereals	Barley	27308	639208
	Oats	2158	36900
	Rye	210	49620
	Triticale	120	15054
	Corn	116955	-
Vegetables and fruits	Potato	16833	-
	Carrot	7344	-
	Onion	4161	-
	Garlic	3474	-
	Fresh green peas	3419	-
	Other vegetables^a^	4915	-
	Apple tree	1454	423
	Pear tree	218	63
	Other fruit trees^b^	48	1216
	Almond tree	610	3010
	Walnut tree	272	356
	Vineyard	3391	58092
	Olive tree	950	2528
Oil seeds	Sunflower	17430	171864
	Rapeseed	2996	13684
Sugar cane & beet	Sugar beet	23864	-

Crops not elsewhere classified	Alfalfa	38031	31253
	Fodder vetch	3400	39336
	Meadows	849	5661
	Other fodders	333	44783
	Green peas	679	33974
	Vetch	1300	30446
	Other peas	1130	9579

a It includes lettuce, zucchini, cabbage, spinach, asparagus, watermelon, melon, tomato, pepper, other.

b It includes apricot tree, cherry tree, peach tree, plum tree.

Agriculture in Castile and León is predominantly rainfed (90%), albeit there has been a significant irrigation expansion over the past decades. This has led to higher production, employment, and added value but has also strained the region's limited water resources. The area of the Castile and León region roughly matches that of the Spanish part of the Douro River Basin (DRB), the Iberian Peninsula's largest basin. The availability of renewable water resources in the region has been decreasing from 14,166.4 million cubic meters/year over 1940-1980 to 12,712.3 million cubic meters/year over 1980–2016. Coupled with growing demand, particularly from agriculture which represents 89.4% of total water uses (3,871.81 million cubic meters), the Water Exploitation Index for the basin has reached 34.1%, well beyond the water-scarce threshold (20%) and nearing severe scarcity (40%). Water shortages are also becoming more frequent and severe, significantly affecting agriculture. To address water scarcity, authorities are exploring demand-side water reallocation policies such as water charges, which aim to reduce withdrawals through higher levies on water use. There is significant research available on the hydrologic and microeconomic impacts of charges and other demand- and supply-side water reallocation policies in the Castile and León region and elsewhere in Spain (Albiac et al., 2020; Pérez-Blanco et al., 2022; Pérez-Blanco and Gutiérrez-Martín, 2017; Sapino et al., 2022b), which show disparate results. On one hand, works using standalone microeconomic models find that water charges can save water at a substantial economic cost, thus limiting its acceptability as a standalone policy instrument (Albiac et al., 2020; Sapino et al., 2020). On the other hand, studies using micro-macro-economic coupled static models suggest that economic costs of water charges may be offset by higher commodity prices due to constrained supplies in relevant markets—albeit these analysis are static and do not consider temporal dynamics (Parrado et al., 2020; Pérez-Blanco et al., 2022).

Agricultural areas in Castile and León and elsewhere Spain are administratively divided into Agricultural Districts (or *comarcas* in Spanish), which incorporate both rainfed and irrigated areas. Irrigated areas can be further subdivided into Agricultural Water Demand Units (AWDUs), which are administrative units that share a common water source and irrigation management practices within the DRB. In the Castile and León Region, there are 60 Agricultural Districts and 200+ AWDUs. Our coupled micro-macro-economic model adopts AWDUs as economic agents of the microeconomic model, which significantly increases granularity as compared to previous studies that relied on agricultural districts as an agent. This has the advantage of allowing for a more detailed assessment of water policies while observing hydrological boundaries and integrity.

## 4 Methodology

This section presents the dynamic micro- and macro-economic coupling method, including a description of the models that populate the modules (PMAUP for the microeconomic module, and CGE for the macroeconomic module), the dynamic coupling to interconnect them, and the experimental design and scenarios used in the simulation. The PMAUP model is implemented in the GAMS language (GAMS, 2019), while the CGE model is developed using GEMPAK (Horridge et al., 2018). The coupling between these models is managed using R (Statistical R Core Team, 2018), which we have chosen as the most suitable option due to its capability to initiate the other two programs and efficiently handle data exchanges during each simulation run. All data inputs are reported in the database available in Annex I (microeconomic model) and Annex II (macroeconomic model) in the supplementary materials.

### 4.1 Microeconomic model

The **PMAUP model** is a mathematical programming model designed to replicate the behavior of economic agents. In agricultural economics, economic agents are either individuals or groups of individuals with common features (e.g., farmers sharing the same water source, as the case in this paper). Mathematical programming models are commonly employed in agricultural economics to simulate the economic impact of changes in the quantity/quality of inputs (such as water availability) or in policies (such as water charging). For an exhaustive review of models used in agricultural economics, readers can refer to Graveline (2016) and Soriano et al. (2025).

PMAUP is a data- and computational-intensive model that advanced significantly in recent decades, driven by the increase in computational power and the availability of novel micro-level data (e.g., farm level production costs, prices, and yields) (Pérez-Blanco et al., 2023). PMAUP is a positive approach, i.e., its parameters are calibrated to minimize the difference between the modeled and observed behavior of agents. Positive models are particularly well-suited for forecasting the impacts of policies or natural shocks against the status quo because the calibrated outcomes closely replicate observed data (Graveline, 2016).

PMAUP is a multi-attribute model, i.e., it explores the relevance of multiple attributes in explaining human behavior, unlike other mathematical programming models in agricultural economics that consider expected profit as the sole attribute influencing farmers' decisions. The general mathematical statement of the PMAUP model is as follows:

[1] $$\mathrm{Max}\;U\left(X\right)=f\left(z_{1}\left(X\right),\ldots ,z_{m}\left(X\right)\right)$$

Subject to:

[2] $$x_{i}\geq 0$$

[3] $$\sum_{i=0}^{n}x_{i}=1$$

[4] $$X\in F\in R^{n}$$

[5] $$z_{1}\left(X\right),\ldots ,z_{m}\left(X\right)=Z\left(X\right)\in R^{m}$$

In equation (1), U(X) represents the utility/objective function, which adopts a Cobb-Douglas specification, following common practice in PMAUP. This objective function is composed of m utility-relevant attributes $z_{1}\left(X\right),\ldots ,z_{m}\left(X\right)$. In our application, we explore the relevance of five relevant attributes (*m=5*), namely: profit; risk; and management complexity, represented through three proxies: total labor, hired labor, and the ratio of direct costs to total income (Gómez-Limón, 2020; Gutiérrez-Martín and Gómez, 2011; Pérez-Blanco et al., 2023). All attributes follow the “more-is-better” rule, i.e., if the provision of one attribute increases and the provision of the remaining attributes remains constant, then total utility increases. “Less-is-better” attributes such as risk or management complexity are thus redefined as avoided risk and management complexity. A description and mathematical statement of the attributes used in the model, of relevance for the coupling procedure, is provided below:

- *Expected profit,* $z_{1}\left(X\right)$

[6] $$z_{1}\left(X\right)=\sum_{i}x_{i}{\bar{\pi }}_{i}-{\sum_{i}x_{i}w}_{i}g$$

Where

[7] $${\bar{\pi }}_{i}=\frac{\sum_{t=1}^{T}\pi_{i,t}}{T}$$

[8] $$\pi_{i,t}=p_{i,t}y_{i,t}+s_{i,t}-c_{i,t}y_{i,t}$$

Where $z_{1}\left(X\right)$ is the attribute expected profit, $x_{i}$ is the land share allocated to each crop *i*, ${\bar{\pi }}_{i}$ is the average gross margin for each crop *i* over the period for which relevant data is available (*t=1, …, T*), $w_{i}$ is the average amount of water use for each crop *i* over the period for which relevant data is available (in m^3^/ha), and g is the water charge (in EUR/m^3^) which in the baseline is set to 0 (but increases in the water charges simulations). The gross margin for each year *t* and crop *i*, or $\pi_{i,t}$, is obtained as price ($p_{i,t}$, in EUR/kg) times yield ($y_{i,t}$, in kg/ha) plus coupled subsidies ($s_{i,t}$, in EUR/ha) minus the variable costs ($c_{i,t}$, in EUR/kg) time yield. Yield ($y_{i,t}$) and prices ($p_{i,t}$) are key variables in the coupling protocols, which render both variables endogenous via inputs from the macroeconomic model. Any change in yield and prices from the macroeconomic model affect attribute $z_{1}\left(X\right)$ constraining the agent to revise the optimization problem in eq. [1] – [5] and thus driving changes in the crop portfolio.

- *Avoided risk,* $z_{2}\left(X\right)$

[9] $$z_{2}\;\left(X\right)={\hat{X}}^{t}VCV\left(\pi \right)\hat{X}-X^{t}VCV\left(\pi \right)X$$

Where $z_{2}\;\left(X\right)$ is the attribute avoided risk, VCV(π) is the variance and covariance matrix of profit, $\hat{X}$ and X are the profit maximizing crop portfolio and an alternative crop portfolio, respectively. Accordingly, the first term in the right-hand side of the equation, ${\hat{X}}^{t}VCV\left(\pi \right)\hat{X}$, quantifies the risk of the risk maximizing crop portfolio, while the second term, $X^{t}VCV\left(\pi \right)X$, quantifies the risk of an alternative crop portfolio.

- *Total labor avoided,* $z_{3}\left(X\right)$

[10] $$z_{3}\;\left(X\right)=\sum_{i}\hat{x_{i}}N_{i}-\sum_{i}x_{i}N_{i}$$

where $z_{3}\;\left(X\right)$ is the attribute total labor (hired and family labor) avoided, the first *proxy* for management complexity avoidance (Bartolini et al., 2007; Sumpsi et al., 1997), $N_{i}$ is the total labor requirements per hectare of crop i, $\hat{x_{i}}$ are the land shares corresponding to a crop portfolio $\hat{X}$ that returns the highest possible total labor, and $x_{i}$ are the land shares corresponding to an alternative crop portfolio X. Accordingly, $z_{3}\left(X\right)$ quantifies the difference between the total labor used in the total-labor maximizing crop portfolio and that of an alternative crop portfolio.

- *Hired labor avoided,* $z_{4}\left(X\right)$

[11] $$z_{4}\left(X\right)=\sum_{i}\hat{x_{i}}H_{i}-\sum_{i}x_{i}H_{i}$$

Where $z_{4}\left(X\right)$ is the attribute hired labor avoided, the second *proxy* for management complexity avoidance (Bartolini et al., 2007; Sumpsi et al., 1997), $H_{i}$ is the hired labor requirements per hectare of crop i, $\hat{x_{i}}$ are the land shares corresponding to a crop portfolio $\hat{X}$ that returns the highest possible hired labor, and $x_{i}$ are the land shares corresponding to an alternative crop portfolio X. Accordingly, $z_{4}\left(X\right)$ quantifies the difference between the hired labor used in the hired-labor maximizing crop portfolio and that of an alternative crop portfolio.

- *Ratio of direct costs to total income,* $z_{5}\left(X\right)$

[12] $$z_{5}\left(X\right)=\sum_{i}\hat{x_{i}}D_{i}-\sum_{i}x_{i}D_{i}$$

Where $z_{5}\left(X\right)$ is the attribute ratio of direct costs and total income, the third proxy for management complexity avoidance (Bartolini et al., 2007; Gutiérrez-Martín and Gómez, 2011), $D_{i}$ is the ratio of direct costs to total income per hectare of crop i, $\hat{x_{i}}$ are the land shares corresponding to a crop portfolio $\hat{X}$ that returns the highest possible ratio of direct cost and total income, and $x_{i}$ are the land shares corresponding to an alternative crop portfolio X. Accordingly, $z_{5}\left(X\right)$ quantifies the difference between the ratio in the portfolio maximizing this ratio and that of an alternative crop portfolio.

In the model, agents aim to maximize U(X) through the choice of a specific crop portfolio, denoted as $X\in R^{n}$, where X is a vector representing the allocation of land to individual crops, $x_{i}$ (i=1,…,n). The crop portfolio choice is constrained by the domain F.

A detailed description of the constraints conforming the domain is available in Annex I in the supplementary material (Section A.I.1). Annex I also includes a description of the calibration procedure (A.I.2), which follows the methodology proposed by Gutiérrez-Martín and Gómez (2011), alongside the data source (A.I.3), calibration results (A.I.4) and the database used to quantify all variables (A.I.5).

### 4.2 Macroeconomic model

The macroeconomic module relies on a regionalized CGE model based on the Global Trade Analysis Project (GTAP) (Hertel, 1997), as described by Parrado et al. (2019, 2020) and Pérez-Blanco et al. (2022). The regionalized database is an extension of the GTAP database at the EU-NUTS^1^ level developed by Bosello and Standardi (2018). The model assumes perfect competition, full employment of production factors, and savings-driven investments. Each region has representative agents for households and firms as part of the economy where markets are cleared by price adjustments to ensure that demand equals supply. The regional and sectoral aggregation of the model is shown in Table 3, with Spain divided in 17 NUTS2 regions. Along these regions, there is one macro region representing the rest of EU27+UK and another representing the rest of the world. Within each region, there are 15 economic sectors, with 8 representative crop categories which have been mapped to the PAUMP model. A description of the supply and demand side of the model and its data sources are available in Annex II.

**Table 3:** Regional and sectoral aggregation of the regionalized CGE model. For the crop categories we identify the corresponding individual crops from the microeconomic model (see Table 2). Source: Parrado et al. (2020).

Regions (s)	Detail	Sectors	Detail
Spain (NUTS 2)	1) Galicia 2) Asturias 3) Cantabria 4) Pais Vasco 5) Navarra 6) La Rioja 7) Aragon 8) Madrid 9) Castilla y Leon 10) Castilla y Mancha 11) Extremadura 12) Cataluña 13) Valencia 14) Baleares 15) Andalucía 16) Murcia 17) Canarias	**Crops**	1) rice 2) wheat 3) other cereals 4) vegetables and fruits 5) oil seeds 6) sugar cane & beet 7) plant based fibers 8) crops not elsewhere classified
Rest of the World	18) Rest of EU-27+UK 19) Rest of the world	**Industry**	9) livestock 10) extraction, fishing and forestry 11) food industry 12) rest of industry
		**Services**	13) utilities 14) construction 15) services

Compared to previous versions by Parrado et al. (2019, 2020) and Pérez-Blanco et al. (2022), the model has been modified to include a recursive dynamic module which accounts for exogenous shifters and capital accumulation allowing for the projection of simulations in future years. The recursive dynamics of the model are mainly governed by two driving mechanisms. The first one regards exogenous trends for primary factors (land and labor), input productivities and policy instruments. These exogenous trends can be used to project a dynamic scenario including policy shifts and global market trends. The second mechanism is the capital accumulation as described in equation (13):

[13] $$K_{t+1}=K_{t}-{\delta K}_{t}+{Inv}_{t}$$

where *K*_*t*_ is the amount of capital available in period t, *δ* is the depreciation rate, and *Inv*_*t*_ represents investments in period t net of depreciation.

### 4.3 Coupling

**Fig. 2:**
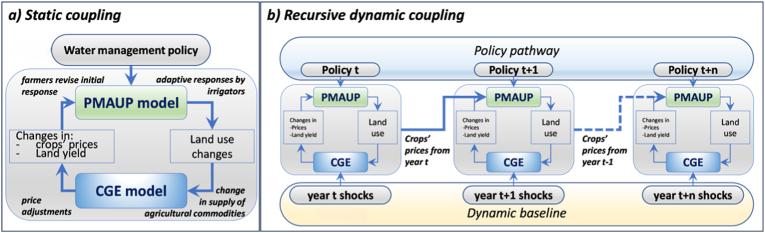
Illustration of (a) Static coupling vs. (b) Dynamic coupling framework. The blue lines indicate the protocols, i.e., information exchanged between the models as part of the coupling. Depending on the modeling scenario, the processes within each box may be repeated in a loop until convergence is achieved—either before advancing to the next year in dynamic simulations or before completing the simulation in static cases.

The coupling between the two models iteratively exchanges time-variant data on land use change (from the microeconomic to the macroeconomic module), crop prices (from the macroeconomic to the microeconomic module), and crop yields changes (driven by exogenous trend shifts and capital accumulation processes; from the macroeconomic to the microeconomic module). The dynamic coupling works in a similar way to the conventional static coupling of Parrado et al. (2020, 2019), with one key difference: while the static version provides a solution for one single period (see panel a) in Fig. 2), the dynamic version iteratively couples the micro- and macro-economic modules over a series of time steps (1 year each) within a dynamic timeframe, where the variables of the coupled model are iteratively updated over time following two-way feedbacks between micro- and macro-economic systems. This makes possible both to assess the impact of exogenous shocks (policy, other), and its evolution over time (e.g., progressive introduction of a policy) alongside endogenous processes including capital accumulation, by passing information of the coupling variables (crop prices, land use, yields) from one year to the next one (see panel b of Fig. 2).

The dynamic model can be resolved in two alternative ways: with and without convergence. Under the convergent design, it is assumed that microeconomic agents have perfect information on the impact of land use change, prices and yields, and thus can anticipate these impacts to optimize the crop portfolio every single year (i.e., the chosen crop portfolio maximizes not only the expected but also the realized utility). In practice, this is implemented by running the micro- and macro-economic simulations iteratively within the same year until convergence is reached, and only then the simulation follows to the next year. Under the non-convergent design, individuals are assumed to be myopic and cannot anticipate the impact of land use change on prices and yields, i.e., they simply react to the *observed* changes in prices and yields over past years. This is done in practice by running the CGE and PMAUP models in sequence, with one single iteration for each year.

The dynamic coupling can be thus understood as a series of static couplings, each one initialized by exogenous shocks and endogenous capital accumulation. The coupling allows for the possibility of assessing policies relying on: i) convergence (bidirectional, iterative feedback), where the model repeatedly exchanges information between agents and markets until results stabilize—reflecting full information on yields and prices; or ii) using a myopic behavior (sequential, single-pass) where only the first iteration of the static coupling is considered, representing an adaptive behavior with limited market information. Critically, to assess the impact of a policy under a dynamic design where conditions are changing over time independently of the shock (due to capital accumulation), the development of a baseline is necessary. The baseline is developed by creating a dynamic simulation without the shock to be studied (in our case, a policy shock). In the baseline simulation, the first signal comes as a change in crops’ prices and yields from the macroeconomic model driven by capital dynamics. These changes trigger a response in the microeconomic model from water users who reallocate land, which in turn feeds the macroeconomic model in the next step of the iterative process. The resultant dynamic baseline simulation represents a situation where there are no policy shocks (e.g., water policy) in place, but the economy is in constant change due to the exchange of information between the micro- and macro-economic models. Once the baseline is ready, the policy (or other shock) can be simulated, including through a dynamic policy pathway, to generate novel outcomes that can be compared to those of the baseline. Both baseline and simulation consider the same constraints that conform the domain (see Section A.I.1 in the supplementary material).

Fig. 2 graphically represents the dynamic coupling (panel b), comparing it to the conventional static coupling (panel a), and presents the coupling protocols.

Below we provide a description and a mathematical statement of the three protocols used for the coupling between the microeconomic PMAUP model and the macroeconomic CGE model, namely: the land use protocol, the crop price protocol, and the yields protocol.

In Stage 1 (**land use protocol**), a policy shock (*g*), in our case a water charge increase, is simulated with the microeconomic PMAUP model and the resulting land use changes transferred to the macroeconomic CGE model. The policy shock affects the utility-relevant attributes described in equations (6)–(12) (in the case of water charges, the attribute directly affected is expected profit in equations (6)–(8)) and triggers a response from the microeconomic agents (AWDUs in this case), who revise their crop portfolio choices by solving the utility maximization problem in equations (1)–(5). This results in a new optimal crop portfolio ${\hat{X}}_{g,p_{0},y_{0}}$, where g, $p_{0}$ and $y_{0}$ refer to the policy shock, crop commodity prices and crop productivity, respectively. The results from the microeconomic simulation allow us to compare land use by each crop *i* before (*g=0,* which leads to the crop portfolio ${\hat{X}}_{0,p_{0},y_{0}}$) and after (${\hat{X}}_{g,p_{0},y_{0}}$) the policy change. Note that in this first step, commodity prices $p_{0}$ and crop productivity $y_{0}$ remain unchanged since the macroeconomic model has not been activated yet—something that will change after Stage 2. Next, land use per individual crop *i* is aggregated into land use per macroeconomic crop category *j* (see Table 2 for a correspondence between individual crops *i* and of crop categories *j*) and region *s* (the relevant spatial disaggregation for the macroeconomic agents). By calculating the ratio between the land use per crop category *j* and region *s* after (${LU}_{g,p_{0},y_{0},j,s}$) and before (${LU}_{0,p_{0},y_{0},j,s}$) the policy change, we can calculate the land use changes per crop category (in percentage), or $\varphi_{gp_{0},y_{0},j,s}$, as shown in equation (14).

[14] $$\varphi_{gp_{0},y_{0},j,s}=\Gamma_{j}\left(\frac{{LU}_{g,p_{0},y_{0},j,s}}{{LU}_{0,p_{0},y_{0},j,s}}-1\right)*100$$

where $\Gamma_{j}$ is the ratio of total land use by the crop category *j* in the AWDUs considered in the microeconomic model to the observed total land use by the crop category *j* in the corresponding region *s* (NUTS2, the agent in the macroeconomic CGE model).

Next, we calculate the land use allocation per crop category *j* and region *s* following the policy shock ($v_{land_{g,p_{0},y_{0},j,s}}$) as a function of the original land use allocation per crop category *j* and region *s* (prior to the policy shock without charges, *g=0*) and the land use changes per crop category obtained in equation (14), and feed it as a shock to the macroeconomic model.

[15] $$v_{land_{g,p_{0},y_{0},j,s}}=F\left(\varphi_{g,p_{0},y_{0},j,s},v_{land_{{0,p}_{0},y_{0},j,s}}\right)$$

For a more detailed description of land allocation in the CGE, the reader is referred to the Supply-side description of the model available in Annex II.

In Stage 2, the CGE runs a simulation with the new land use allocations obtained in equation (15) and finds updated commodity prices ($p_{1}$) **(crop price protocol**) and crop productivities ($y_{1}$) **(yields protocol**), for each crop category *j* and region *s*. Crop productivity is defined as the percentage change of the ratio between the total output (${Prod}_{j,s}$) and land use per crop category j and region s (${LU}_{j,s}$). Note that the key driver of crop productivity change is capital accumulation (see equation (13)).

[16] $$y_{j,s}=F\left({Prod}_{j,s}/{LU}_{j,s}\right)$$

In Stage 3, both $p_{1}$ and $y_{1}$ are conveyed from the macroeconomic to the microeconomic model. This is done by adjusting the price and yield per individual crop i and AWDU in equation (6) (expected profit attribute) in accordance with the price and crop productivity changes of the corresponding macroeconomic crop category *j* and region *s*. Subsequently, a simulation is run where the microeconomic PMAUP model solves the maximization problem stated in equations [1], [2], [3], [4], [5]) to obtain a new crop portfolio ${\hat{X}}_{g,p_{1},y_{1}}$. At this point, a new iteration of the coupling process starts by initiating the land use protocol. Note that in this Stage it is also possible to update the exogenous policy shock *g* if the option for a policy pathway is chosen.

[17] $$\varphi_{gp_{1},y_{1},j,s}=\Gamma_{j}\left(\frac{{LU}_{g,p_{1},y_{1},j,s}}{{LU}_{g,p_{0},y_{0},j,s}}-1\right)*100$$

[18] $$v_{land_{g,p_{1},y_{1},j,s}}=F\left(\varphi_{g,p_{1},y_{1},j,s},v_{land_{{g,p}_{0},y_{0},j,s}}\right)$$

### 4.4 Experimental design and scenarios

Simulations are run using four alternative experimental designs for model coupling: two dynamic coupling designs using the methods described above—Myopic Dynamic Coupling and Perfect Information Dynamic Coupling—and two conventional static designs developed in previous work by the authors (Parrado et al., 2020). By comparing the results using the novel dynamic v. Conventional static coupling we highlight the importance of considering the time dimension when assessing micro-macro-economic systems dynamics and policy impacts. The four experimental designs used for model coupling include:

• **Myopic dynamic coupling (MDC)**: Iterative dynamic design performing one full iteration each year without aiming for convergence. This simulation is designed to represent a myopic-adaptive time-variant behavior within the coupled system, where microeconomic economic agents cannot anticipate the consequences of their actions at the macro level (in other words, farmers take their crop portfolio choices using information on prices, yields and costs observed in previous years). Under the MDC design, after the conclusion of stage 2 in each iteration, a new year in the simulation commences.
• **Perfect information dynamic coupling (DC)**: Similar to the MDC, but with the iterative coupling process continuing until convergence is achieved within each simulation year. The DC assumes that microeconomic agents have perfect information on the impacts their choices have on crop prices, yields, and costs, and can exploit this foresight to maximize their utility (in other words, farmers can anticipate the consequences of their crop portfolio choices on prices as well as the evolution of yields and costs, and adjust their choices accordingly). Under the DC design, stages 1 to 3 are repeated iteratively within each year until convergence is achieved (i.e., crop portfolios in both micro and macro models are stable). Once convergence is achieved, the simulation moves to the next year and repeats the process.
• **No coupling (static)**: Under this model design the microeconomic module operates independently, without any coupling with the macroeconomic module. In other words, the results show the simulation outcomes obtained with the PMAUP model only. This design reflects the traditional way of conducting impact assessments of water scarcity and water (re)allocation policies (Sapino et al., 2022b).
• **Static coupling**: This model design follows the conventional micro-macro-economic coupling approach typically adopted in the literature. Specifically, we adopt the static coupling approach of Parrado et al. (2020), which exchanges information of commodity prices (from the macro-to the micro-economic model) and land use change (from the micro-to the macro-economic model) in a time-invariant fashion. The model iteratively runs the microeconomic and macroeconomic models until convergence is reached, all within a single period (i.e., there is no capital accumulation and no possibility to update water charges).

To correctly attribute the policy impacts under a changing dynamic design, we run simulations for each model above under three policy scenarios: a baseline (or reference) scenario and two water charge scenarios. It is important to note that static models only allow for a single-period policy scenario. By comparing the simulation results of the baseline and policy scenario for each model, we can identify what impacts can be attributed to policy change and distinguish them from other model processes such as exogenous market trends or endogenous capital accumulation and productivity growth. The baseline and water charge scenarios are defined as follows:

⁃ **Baseline scenario,** where there is no water charge policy. In the cases of static and no-coupling methods, the baseline refers to the initial condition with no additional water charge. In the case of the dynamic couplings, the baseline is produced considering GDP and population projections of the Shared Socioeconomic Pathways - SSP2 scenario (O'Neill et al., 2014), adjusted for the 17 Spanish NUTS2 regions plus the rest of the world, using data available at the SSP database (Gidden et al., 2019), and for a period of 12 years starting in 2007 which is the base year of the CGE model. The choice of this period is arbitrary with the only aim of providing a reasonable period for results comparisons.
⁃ **Single Water Charge (SWC) scenario**, where we run a simulation in which we increase the water charge across all AWDUs within the Castile and León Region in Stage 1 of the coupling process by 0.10 EUR/m^3^ (g=0.1). Note that the no coupling model runs only the microeconomic simulation in Stage 1, without conveying any data to the macroeconomic model. Dynamic models simulate the same 12 years of the baseline scenario.
⁃ **Incremental Water Charge (IWC) scenario**, where we run a simulation in which the water charge is gradually increased by 0.10 EUR/m^3^ (g=0.1) in increments of 0.01 EUR/m^3^ across all AWDUs within the Castile and León Region in Stage 1 of the coupling process. This policy can be simulated only with the MDC and DC, the only dynamic models, over the same 12 years of the baseline scenario.

## 5 Results and discussion

### 5.1 Baseline scenario

**Fig. 3:**
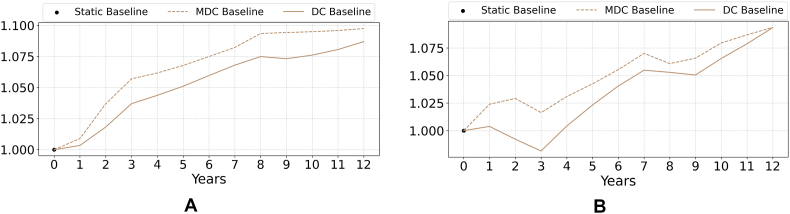
(a.) price index and (b.) yield index in the baseline scenario. See footnote 3 for details on the calculation. Note that these indices are dimensionless.

This section presents the simulation results obtained for the baseline scenario under the alternative experimental designs for the model coupling. Fig. 3 shows prices and yield productivity changes for all model couplings through a price and yield index.^2^ For the static model couplings where the only driver of change is the exogenous policy shock, prices do not change (yield productivity does not change either, since it can only be simulated in the dynamic coupling designs). For the dynamic coupling designs, both price and yield indices increase over time in the baseline, but at different rates: prices grow over the first 10 years, and then become more stable; while yield shows a more continued growth driven by the recursive dynamics process of the CGE model. The decrease in yield in the second and third years reflects the effects of the financial crisis of 2008-2010. Both MDC and DC designs show similar trends, albeit increases in the price and yield indices are consistently higher in the DC.

**Fig. 4:**
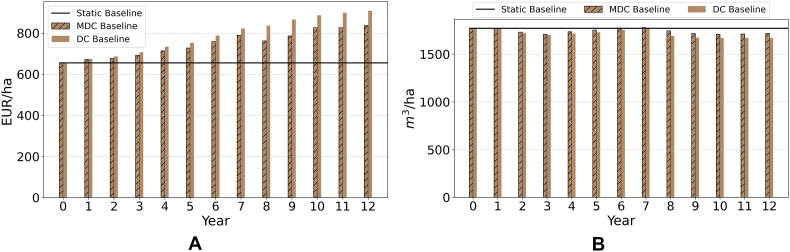
(a.) farmers' average profit (EUR/ha) and (b.) average water use (m^3^/ha) in the baseline over time.

Fig. 4 shows changes in relative profit and water use per hectare in the baseline. For the dynamic coupling experimental design, agricultural profit per hectare consistently increases for both the MDC and DC over the simulation period (Fig. 4a), albeit there is a widening gap that can be attributed to the higher commodity prices reported for the DC design in Fig. 3. This finding is consistent with the assumption of perfect information playing in favor of higher returns for economic agents. Water use per hectare (Fig. 4b) remains nearly constant over time, with a slight decrease attributable to gains in crop productivity as well as changes in crop prices (see Fig. 3), which drive the adoption of less water-intensive crops while increasing profit. Again, for the static model design where the only driver of change is the exogenous policy shock, neither profit nor water use change, differently from the dynamic designs where the time dimension introduces more feedback mechanisms at play as described in Fig. 2.

### 5.2 Policy impacts (baseline v. water charge scenarios) and discussion

This section reports the simulation of the water charge v. Baseline scenarios under alternative experimental designs for the model coupling, namely the conventional “no coupling” (static microeconomic model as in Sapino et al. (2022)) and micro-macro-economic “static coupling” (Parrado et al., 2020) and the proposed dynamic micro-macro-economic couplings (“MDC” and “DC”). The purpose is to illustrate the performance of dynamic v. Static models and highlight relevant differences in simulation outcomes. We compare the results obtained in the SWC and IWC scenarios with those obtained in the baseline scenario to isolate the impacts of the water charging policy under each model coupling design. The objective is to separate the effects of the policy shock from those originating from the endogenous dynamics of the modeling framework (e.g., due to capital accumulation). It should be noted that all model coupling designs share the same initial conditions in year 0—i.e., water charge and baseline scenarios yield the same results. This initial condition is indicated across all figures with a black point. Since the “no coupling” (Sapino et al., 2022b) and “static coupling” (Parrado et al., 2020) model designs are time-invariant, they jump from the initial condition in year 0 to the policy scenario simulation also in year 0, and results are assumed to remain constant afterwards.

**Fig. 5:**
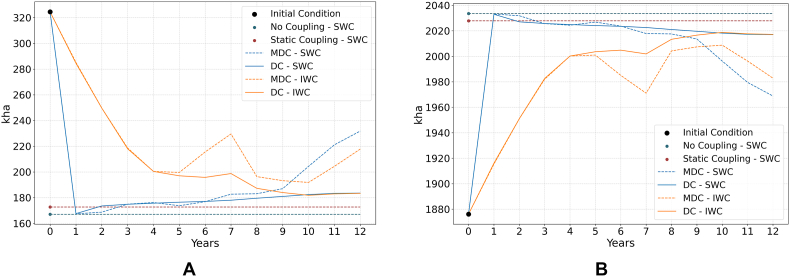
Land use [in 1000 ha] for irrigated (a.) and rainfed (b.) crops under the SWC and IWC scenarios. Static designs provide an atemporal response, which is shown in year 0 and projected onto subsequent years for comparison purposes.

Fig. 5 presents the impact of water charge scenarios (SWC, IWC) on land use in the Castile and Leon Region over time for the alternative model coupling experimental designs. Results in Fig. 5 are aggregated for rainfed and irrigated crops, with detailed results for each crop category *j* available in Annex III. Higher water charges produce an abrupt substitution of irrigated by rainfed crops across all water charge scenarios (SWC, IWC) and model coupling designs, with the “no coupling” model (Sapino et al., 2022b) displaying the most significant shift towards rainfed crops, followed by the dynamic and static (Parrado et al., 2020) coupling. The more abrupt shifts observed under the “no coupling” (Sapino et al., 2022b) as compared to the coupled (dynamic and static) models is due to the price feedback: higher water charges in the coupled models reduce the economic return, surface and supply of irrigated crops, which in turn increases the prices and economic return of irrigated crops, making irrigated crops more appealing and thus mitigating the initial reduction in their surface as compared to the “no coupling” model (Sapino et al., 2022b).

Regarding the transition dynamics under the alternative water charge scenarios (dynamic couplings MDC and DC only), the SWC scenario produces an abrupt substitution of irrigated by rainfed crops in year 1 for both MDC and DC model couplings, followed by a gradual recovery of the surface of irrigated crops over the next 9 years of simulation (with a smoother trend observed for the DC model). From year 10 onwards, the MDC shows a pronounced increase (decrease) of irrigated (rainfed) crops, while in the DC model design the land use of both rainfed and irrigated crops stabilizes. This is consistent with the convergent design of the DC model, which tends to produce more stable crop portfolios as compared to the MDC design. Under the IWC scenario, a rapid substitution (albeit less abrupt than in the SWC) of crops during the first five years is followed by a more gradual transition in the final five years—suggesting that the most price-sensitive crops are replaced within the initial five years of the adjustment process.

**Fig. 6:**
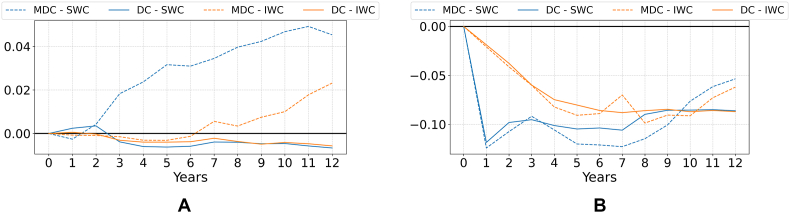
Changes in the (a.) price index and (b.) yield index, measured as the difference between the indices value in the SWC and IWC scenarios v. The baseline scenario. Price and yield indices are dimensionless.

Fig. 6 reports the differences between the price and yield indices in the water charge (SWC, IWC) v. Baseline scenario, for dynamic (MDC, DC) and static model couplings. A value higher (lower) than 0 in Fig. 6 indicates that prices/yield productivity in the SWC and/or IWC scenario are higher (lower) than those of the baseline scenario (price and yield indices in the baseline scenario are reported in Fig. 3). Note that prices and yield productivity in the SWC and IWC scenarios display a growing trend, as they do in the baseline scenario in Fig. 3; what we are showing in Fig. 6 is the difference between prices and yield productivity in the SWC and IWC v. Baseline scenario. Also note that in the “no coupling” model (Sapino et al., 2022b) prices are exogenous, and thus this model is not represented in the price and yield figure. Finally, since the yield index emerges from endogenous and time-variant capital accumulation processes, it is not reported for the static coupling design (Parrado et al., 2020).

Regarding prices (Fig. 6a), the impact of the SWC scenario is ambiguous and can both increase or reduce commodity prices relative to the baseline scenario, depending on the model and the time horizon considered. The static coupling design (SWC) (Parrado et al., 2020) shows a one-off 1% price increase relative to the baseline following the water charge increase, due to the reduced surface of irrigated crops which constrains the overall supply of agricultural commodities (rainfed is less productive than irrigated agriculture). The MDC design shows lower prices in the SWC scenario than in the baseline scenario for year 1 of the simulation, but from year 2 the situation reverts with commodity prices in the water charge scenarios becoming increasingly larger than those in the baseline scenario until year 11—where the gap starts decreasing. For the IWC scenario, the MDC follows a similar pattern than in the SWC scenario but with a delayed response due to the incremental water charge. Commodity prices are lower than the baseline until year 6, after which prices consistently increase. In the DC model design, the situation is the opposite: prices in the SWC scenario are higher than those of the baseline scenario in year 1 and 2, but from year 3 the situation reverts, and the baseline scenario prices are consistently higher than the policy scenario prices, albeit this difference is small and stable. Under the IWC scenario, a similar trajectory is observed but prices exceed those of the baseline only in the first year. This difference between the DC and MDC models is explained by the convergent design of the DC, which reduces inter-year price fluctuations.

Regarding yield productivity (Fig. 6b—only relevant for dynamic couplings), the SWC scenario triggers a similar response from the MDC and DC, with the former showing more abrupt changes and the latter exhibiting a more stable trend consistent with its convergent design. Under the IWC scenario, MDC and DC both show a reduction in yield, though less abrupt than under the SWC. DC model forecasts eventually stabilize at the same levels for both SWC and IWC from year 9 onwards, while the DWC model forecasts exhibit more pronounced fluctuations and a growing trend in the final years of the simulation.

**Fig. 7:**
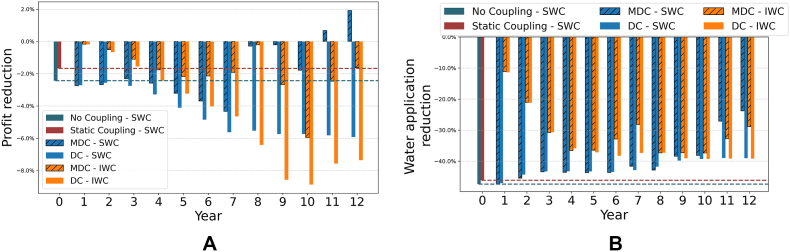
Changes in (a.) profit and (b.) water use, measured as the difference between their values in the SWC and IWC scenarios v. Baseline scenario.

Fig. 7 shows the change in agricultural profit (Fig. 7a) and water use (Fig. 7b), measured as the percentage change of these variables in the water charge (SWC, IWC) scenario relative to the values in the baseline scenario. The “no coupling” design (Sapino et al., 2022b) shows the highest impact both in terms of water use (−47%) and profit (−2.43%) in the SWC as compared to the baseline scenario. These results overestimate the impact of water charges on profit and water use by ignoring the mitigating effect of economy-wide feedbacks through higher commodity prices and yields (Parrado et al., 2020). When the macroeconomic feedback is considered in the static coupling design (Parrado et al., 2020), results show slightly smaller water savings and a significantly smaller (1.67%) reduction in profit.

Also under the SWC scenario, the MDC design generates significant water savings initially as compared to the baseline, but water use significantly increases subsequently (from an initial water use change of −47% in year 1 to −24% in the last year of the simulation). The DC design displays a significant and similar reduction in water use initially, followed by a slight increase in water use over the following periods (from an initial water use change of −47% in year 1 to −39% in the last year of the simulation). These results suggest a decreasing effectiveness of the SWC scenario in reducing water use under time-variant designs. Under the IWC scenario, both the MDC and DC couplings show progressive and sustained reductions in water use until year 5. After this point, the MDC shows some instability, peaking in year 8 at −37.39% water use change, before water use starts to increase until −29% in the last year of the simulation. The DC reaches its peak water use reduction in year 10 at −39.3%, and then remains relatively stable.

Regarding profit, under the SWC scenario the MDC reproduces a cyclical behavior where the impact of the policy shock on profits as compared to the baseline scenario is first significant and negative, then negative and smaller, then negative and significant again, and finally positive and significant (i.e., profits are initially more volatile and lower in the SWC as compared to the baseline scenario, but eventually become larger). The DC design, conversely, shows a consistent reduction in profit over time as compared to the baseline scenario, which tends to stabilize during the last years of the simulation. Under the IWC scenario, both the MDC and DC show a progressive reduction in profit compared to the baseline scenario until year 5. From year 6 onward, the DC continues this trend, while the MDC begins to show fluctuations like those under the SWC but without reaching a positive value (i.e., profit under the SWC is consistently lower than that of the baseline). The difference between SWC and IWC simulations can be attributed to the continuous increase in water charges under the IWC, which slows down adaptation compared to the SWC scenario. Instead of allowing a quick adjustment, the gradual price increase delays the price effect, resulting in a slower recovery. Again, the relatively more stable simulation outcomes obtained under DC reflect on the convergent nature of this model design, which leads to consistent trends rather than cyclical outcomes as those observed in the MDC. Again, it is important to note that negative profit values in Fig. 7 only reflect a lower profit value in the water charge scenarios as compared to the baseline, while total profit is positive and growing from year 1 to 12. Additionally, the higher profit reduction observed in the DC model is partly explained by the higher profit values in the DC Baseline (see Fig. 4a).

## 6 Conclusions

This study dynamically couples a micro- and macro-economic model to assess the time-variant impacts of environmental policy. The dynamic coupling addresses conventional static model coupling limitations by iteratively exchanging time-variant data on land use change (from the microeconomic to the macroeconomic module), crop prices (from the macroeconomic to the microeconomic module), and crop yields changes (driven by exogenous trend shifts and capital accumulation processes; from the macroeconomic to the microeconomic module), while also increasing spatial granularity. This enables a more detailed assessment of farmers’ adaptive behavior and market feedback under nonstationary/changing conditions like climate change or sequential policies. We illustrate our methodology with an application to agricultural water charges—applied both as a one-time (SWC) and incremental (IWC) adjustments—in the Castile and Leon Region (Spain) under four alternative model couplings, namely, two novel dynamic micro-macro-economic model couplings and two conventional static model designs. Our results show that water charge scenarios under the dynamic coupling reveal a gradual recovery in water use over time after the implementation of the SWC, thus significantly reducing the policy effectiveness suggested by static coupling results.

Our approach, while insightful, has limitations that necessitate further research. *Firstly*, the integration of recent advancements in microeconomic modeling can improve the overall framework. Recent developments have decoupled land use choices from water use decisions, suggesting that crop portfolio choices are not linearly tied to water use as it is typically assumed in microeconomic models (Graveline and Mérel, 2014; Sapino et al., 2022a). This allows for more realistic representations of adaptive responses such as deficit or supplementary irrigation (Koundouri, 2004). Another limitation of the current microeconomic model is its inability to integrate multi-annual decisions, such as investments in new technologies (e.g., efficient irrigation systems, adoption of new crops, or adjustments in the area allocated to permanent crops). On the other hand, integrating more complex models can significantly increase computational costs and require additional data that may not be readily available. For these reasons, the current version of the model does not include these options. However, as data availability and computational capacity improve in the future, integrating more advanced modeling techniques can become a viable and potentially beneficial addition to our approach.

*Secondly*, it is necessary to address uncertainty in the modeling framework to produce more comprehensive simulation outcomes, and thus inform robust policies that provide a satisfactory performance under most plausible futures (Marchau et al., 2019). Yet, there is no “free food for robustness” (Hino and Hall, 2017), and addressing the relevant sources of uncertainty (in data inputs, parameters calibration, and the definition of the structure of the model) necessitates techniques such as multi-model ensembles (structural uncertainty) and global sensitivity analysis (input and parameter uncertainty) whose computational cost increases exponentially with the number of model inputs—which is particularly large in the case of coupled models. The use of emulators that apply machine learning techniques to develop metamodels that can thoroughly quantify uncertainty at a significantly lower computational cost could be explored to address this limitation (Saltelli et al., 2020).

*Thirdly*, the integration of a hydrological model could help us to better understand the environmental implications of the policy shock and its socioeconomic impacts, in line with recommendations from the literature on socio-ecological systems and socio-hydrology modeling (Sivapalan et al., 2012). Notably, the addition of a hydrological model would make possible to assess the impacts of climate change on the water cycle, as well as the economic system (e.g., by translating impacts on the water cycle predicted the hydrological model into more constraining water allocations in the microeconomic model—see equation (4)). Note that adding new system models would further amplify the cost of modeling including uncertainty quantification, potentially conflicting with the second point above.

*Fourthly*, the macroeconomic model could be improved by differentiating between irrigated and rainfed crops across all crop categories *j* (Table 2), as well as by increasing the number of crop categories *j* (ideally to the point where the crop categories *j* in the macroeconomic model match the individual crops *i* in the microeconomic model). This would allow for a more accurate representation of the microeconomic crop portfolio into the macroeconomic model, and enhance the accuracy of our results.

*Finally*, micro- and macro-economic models are calibrated but not validated due to data limitations that hinder proper validation. The relevant economic data required for validation are either unavailable (insufficient historical records) or only accessible at spatial scales (national/regional) that are too coarse for meaningful validation at the necessary resolution. Although this is common practice in the literature and some researchers do not consider this a shortcoming (Cortignani and Dono (2020) argue that positive microeconomic models can be “calibrated and validated by way [of a] positive approach”), we acknowledge the need for researchers to develop a standardized methodology for assessing the forecasting performance of these models. Such an approach would enhance accuracy and facilitate the selection of the most suitable models for each specific context.

## CRediT authorship contribution statement

**Francesco Sapino:** Writing – review & editing, Writing – original draft, Visualization, Validation, Software, Methodology, Formal analysis, Data curation, Conceptualization. **Ramiro Parrado:** Writing – review & editing, Supervision, Software, Methodology, Formal analysis, Conceptualization. **C. Dionisio Pérez-Blanco:** Writing – review & editing, Writing – original draft, Supervision, Project administration, Methodology, Funding acquisition, Conceptualization.

## Declaration of competing interest

The authors declare that they have no known competing financial interests or personal relationships that could have appeared to influence the work reported in this paper.

## Data Availability

Data will be made available on request.

## Footnotes

1 The Nomenclature des Unités Territoriales Statistiques (NUTS), or nomenclature of territorial units for statistics, is “a hierarchical system for dividing up the economic territory of the EU” (EUROSTAT, 2020).

2 To calculate the price index for agricultural products over time, we first multiply the price $p_{i}$, yield $y_{i}$, and surface area $x_{i}$ for each crop i in each year using current prices and then aggregate these values across all crops to obtain the annual total agricultural value at current prices. We then repeat this calculation using constant prices (base year is the first year in the series). Finally, the price index for each year is calculated by dividing the total agricultural value of that year at current prices by the total agricultural value of that year at constant prices.

$$price\;index\;of\;year\;n=\frac{\sum_{i}p_{i,n}\times y_{i,n}\times x_{i,n}}{\sum_{i}p_{i,0}\times y_{i,n}\times x_{i,n}}$$

To calculate the yield index, we multiply the yield, surface area, and price (constant prices from the base year/first year in the series) for each crop, and aggregate the resultant values across all crops to obtain the annual total agricultural value at constant prices. The yield index for each year is then calculated by dividing the total agricultural value at constant prices for that year by the total agricultural value at constant prices of the base year.

$$yield\;index\;of\;year\;n=\frac{\sum_{i}p_{i,0}\times y_{i,n}\times x_{i,n}}{\sum_{i}p_{i,0}\times y_{i,0}\times x_{i,n}}$$
