# Supplementary material for: A dynamic micro-macro-economic model to assess water charging policies
Source: J Clean Prod. 2026 Mar 1;547:147802. doi: 10.1016/j.jclepro.2026.147802 (PMC12935697; doi:10.1016/j.jclepro.2026.147802)
Supplement: Multimedia component 1 [file mmc1.docx]

#### **Annex I: PMAMP model**

#### **AI.1. domain**

The set of constraints that conform the domain $F$ used in the calibration and simulation of the model is described in the following paragraphs.

*Land availability.* Available agricultural land is assumed constant and equals the summation of observed agricultural land uses in the calibration year.

*Water availability.* It is assumed that water abstraction licenses remain constant before and after every simulation run, i.e.:

| $\sum_{i=1}^{n} {w_{i}x}_{i}\leq W$ | (A.I.1) |
| --- | --- |

where $w_{i}$ is crop $i$’s specific water requirements and $W$ is the total water allotment in the study area.

*Climate and soil.* Since each agricultural area/climatic region has its soil and climatic characteristics, agents in the model can only grow those crops that are observable in the database (Essenfelder et al., 2018).

| $\sum_{i=1}^{n} {y_{i}x}_{i}=0 \vert y_{i}\in\{0,1\}$ | (A.I.2) |
| --- | --- |

where $y_{i}=0$ means the crop is observable and $y_{i}=1$ means the crop is not observable in the area.

*Crop-specific constraints.* Some crops in the portfolio have an upper and/or lower area bound because of specific policy restrictions.

| \| $\varphi_{i}x_{i}\leq\left( 1+b_{i} \right)x_{i}^{0} \vert\varphi_{i}\in\left\{ 0,1 \right\}; 0\leq b_{i}\leq1$ \| (A.I.3) \| \| --- \| --- \| |  |
| --- | --- | --- | --- |
|  |  |

Where $\varphi_{i}$ is a binary vector that (de)activates the constraint, $b_{i}$ is the upper bound set (in percentage) and $x_{i}^{0}$ is the *observed* share of land devoted to crop $i$. Equation A.I.3 refers to the upper bound constraint; in the lower bound, the inequality would be the opposite and the right-hand side of the equation would be a subtraction. This restriction could be used to set a minimum/maximum threshold for ligneous trees of ±5%, to prevent large (dis)investments with potentially large impacts on e.g. carbon sequestration, whose economic value is not accounted for in the models, which focus on yearly market variables (notably profit) (Essenfelder et al., 2018).

*Crop rotation.* If two crops rotate with each other, replacing one of them with a third crop that is not compatible with the rotation system implies the removal of the two original crops, i.e.:

| $\sum_{i,j} g_{i,j}x_{i}\leq\sum_{i,j} h_{i,j}x_{i}^{0} \vert g_{i,j}\in\left\{ 0,1 \right\}; h_{i,j}\in\{0,1\}$ | (A.I.4) |
| --- | --- |

where $g_{i,j}$ and $h_{i,j}$ are binary vectors that (de)activate the constraint for every specific crop.

*Technological improvement.* Technological improvements, such as advancements in irrigation efficiency or new agricultural techniques, are assumed to be unavailable or not feasible.

#### **AI.2 calibration procedure**

The **calibration** of the PMAMP model aims to elicit an objective function that is consistent with $\boldsymbol{X}^{o}$ and the choice domain $F$. This model makes calibration possible eliciting the parameters of a non-linear Cobb-Douglas utility function, the arguments of which are competing attributes (e.g., profits v. avoided management complexity). The Cobb-Douglas utility function of a PMAMP model is represented in the next equation (A.I.5):

| $U\left( x \right)= \prod_{p=1}^{5} z_{p}^{\alpha_{p}}(\boldsymbol{X})$ | (A.I.5) |
| --- | --- |

Following the standard economic theory, the parameters of the objective function can be revealed for every possible combination of attributes within a finite set by means of equalizing the opportunity cost of trading one unit of attribute $z_{p}$ off for one unit of attribute $z_{k}$, i.e. the slope of the efficient frontier or Marginal Rate of Transformation (${MRT}_{pk}$), and the willingness to give up one unit of attribute $z_{p}$ in exchange for a unit of attribute $z_{k}$, i.e. the slope of the indifference curve of the utility function or Marginal Rate of Substitution (${MRS}_{pk}$).

This is done in three steps:

1) we use numerical methods to reveal the efficiency frontier for each pair of attributes, and estimate the ${MRT}_{pk}$;

2) we equalize the ${MRT}_{pk}$ and ${MRS}_{pk}$ for a given functional form to elicit the parameters of the objective function for every possible combination of attributes within a finite set;

3) the relevant attributes and the corresponding parameters are those that minimize the distance between observed and simulated decisions.

The rationale of agent’s decisions is explained by the provision of utility-relevant attributes; in turn, the relevance each attribute has on agents’ decisions (i.e. objective function parameters) is determined by agents’ attitudes (Läpple and Kelley, 2013; Poppenborg and Koellner, 2013). Following the axioms of revealed preference, it is possible to calibrate a utility function that captures these attitudes or preferences from observed agents’ choices and the resultant provision of attributes. In doing so, the PMAMP model follows a positive approach, i.e., the optimal solution to the problem above should match or at least be as close as possible to the observed crop portfolio $\boldsymbol{X}^{o}$.

The efficient frontier represents the maximum value of the attribute $z_{p}\left( \boldsymbol{X} \right)$ the economic agent can attain for a given value of the attribute $z_{k}\left( \boldsymbol{X} \right)$ (*p*$\neq k$) within the space of feasible decisions $F$. Real-life efficient frontiers cannot be analytically defined using a closed function. Instead, numerical methods through optimization procedures are typically used to elicit the efficient frontier, such as the constraint method (Marglin, 1967) where all but one objective are transformed to constraints with changing right-hand side values which represent different target values or achievement levels within the feasible range for each attribute. Following the constraint method, we obtain an efficient frontier in the two-dimensional space for each pair of attributes $z_{p}$ and $z_{k}$, which can be defined as $\tau_{z_{p},z_{k}}\left( \boldsymbol{X}^{\boldsymbol{**}} \right)$, where $\boldsymbol{X}^{\mathbf{**}}$ is the set of crop choices delivering a provision of attributes $z_{p},z_{k}$ *along* the frontier. Convexity holds, i.e. increasing the provision of a given attribute will result in the reduction of another attribute; if there is no tradeoff, the choice becomes irrelevant and one of the attributes can be discarded.

Once the efficient frontier has been elicited, a tangency or “landing point” at which the ${MRT}_{pk}$ equals the ${MRS}_{pk}$ has to be found, for which several alternatives exist (Amador et al., 1998; André et al., 2010; André and Riesgo, 2007; Gutiérrez-Martín and Gómez, 2011; Sumpsi et al., 1997). A comprehensive discussion of these alternatives is available e.g. in (Gómez-Limón et al., 2016). The following method was proposed by Gutiérrez-Martín and Gómez (2011) who, for the illustrative case of two attributes, obtain the maximum feasible value of the attribute $z_{p}$ for the *observed* value of $z_{k}$ ($z_{k}=z_{k}^{o}$), and vice versa (figure 1 – case 3). Through the projection of the observed crop portfolio $\left( \boldsymbol{X}^{\mathbf{o}} \right)$ to the efficient frontier this method yields two points, namely $\tau_{z_{p},z_{k}^{o}}$ and $\tau_{z_{p}^{o},z_{k}}$ (points $A$and $B$in in Figure A.1), which can be connected using a hyper-plane to approximate the efficient frontier.


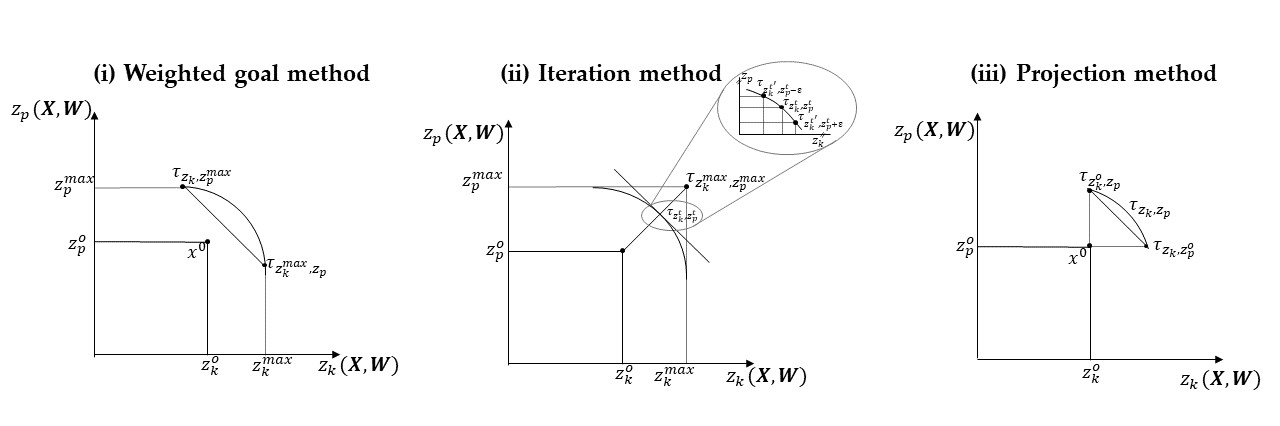


Figure A.1: projection method for the clibration of the PMAUP model

The ${MRT}_{pk}$ is the slope of the segment connecting the two projected efficient points, $\tau_{z_{p},z_{k}^{o}}$ and $\tau_{z_{p}^{o},z_{k}}$:

${MRT}_{pk}=\beta_{pk}=\frac{z_{k}-z_{k}^{o}}{{z_{p}-z}_{p}^{o}}$ (A.I.6)

The slope $\beta_{pk}^{\tau}$ approximates the marginal opportunity cost of trading attribute $z_{p}$ off for attribute $z_{k}$, or ${MRT}_{pk}$; any point along the hyperplane A-B, including points A and B, can be used as a possible tangency point for the calibration of the utility function. Note that since efficient frontiers are convex, the hyper-plane connecting efficient points does not reflect the precise slope of the efficient frontier at points $A$and $B$, leading to possible errors.

The slope of the indifference curve of the utility function, or Marginal Rate of Substitution (${MRS}_{pk}$), is obtained as follows:

| ${MRS}_{pk}=-\frac{\frac{\partial U}{\partial z_{k}}}{\frac{\partial U}{\partial z_{p}}}=-\frac{\alpha_{k}}{\alpha_{p}}\frac{z_{p}}{z_{k}}; p,k\in\mathbb{R}^{m};\forall p\neq k$ | (A.I.7) |
| --- | --- |

Several valid forms can be explored for the utility function.

In this application, the PMAMP model adopts a homothetic Cobb-Douglas functional form. As compared to alternative additive or multiplicative-additive specifications, a Cobb-Douglas function offers the advantages of decreasing marginal utility for each attribute and the existence of a global optimum (Inada, 1963), and provides a sensible approximation to actual farmers’ behavior (Sampson, 1999). By means of equalizing the ${MRS}_{pk}$ of a Cobb-Douglas utility function (A.I.5) to the ${MRT}_{pk}$ obtained in the previous section, the objective function parameters are found for every possible combination of attributes within the finite attribute set considered:

${MRS}_{kp}=- \frac{\frac{\partial U}{\partial z_{p}}}{\frac{\partial U}{z_{k}}}=-\frac{\alpha_{p}}{\alpha_{k}}\frac{z_{k}}{z_{p}}={MRT}_{kp}$ (A.I.8)

$\sum_{p=1}^{m} \alpha_{p}=1$ (A.I.9)

Now it is possible to calculate the values for the utility function parameters $\alpha_{p}$ for every possible combination of attributes as follows:

| $\alpha_{p}=\frac{1}{1-\frac{\sum_{k=1}^{m} \beta_{pk}\cdot z_{k}}{z_{p}}} \forall p\neq k$ | (A.I.10) |
| --- | --- |

Finally, it is possible to calculate the calibration residuals, which provide valuable information to assess model performance. Our application considers three calibration residuals. The first one is obtained as the difference between observed and calibrated crop portfolios (Gutiérrez-Martín and Gómez, 2011):

| $e_{d}=\frac{\sqrt{\sum_{i=1}^{n} \left( x_{i}^{o}-x_{i}^{*} \right)^{2}}}{\sqrt{(\sum_{i=1}^{n} \left( x_{i}^{o} \right)^{2})+1)}}$ | (A.I.11) |
| --- | --- |

where $x_{i}^{*}$ is the fraction of land allotted to crop $i$ in the utility-maximizing crop portfolio $X^{*}$.

The second residual is obtained as the difference between the provision of attributes that is observed and the provision of attributes in the calibrated model:

| $e_{a}=\sqrt{\frac{1}{m}\sum_{p=1}^{m} \left( \frac{z_{p}^{o}-z_{p}^{*}}{z_{p}^{0}} \right)^{2}}$ | (A.I.12) |
| --- | --- |

where $z_{p}^{*}$ is the value adopted by attribute $p$ for the case of the utility-maximizing crop portfolio $X^{*}$.

Following Gutiérrez-Martín and Gómez (2011), the average residual is obtained as:

| $e_{m}=\frac{\sqrt{e_{x}^{2}+e_{\tau}^{2}}}{2}$ | (A.I.13) |
| --- | --- |

#### **AI.3 data**

Table A.I.1 summarizes the data source and reference year of the input data used to measure and quantify the provision of utility-relevant attributes. The **PMAMP model** was calibrated in the year 2015, the last year with available economic data and without water scarcity.

*Table A.I.1. Model inputs and related data providers*

| Variable | Abbreviation used | Data provider | Ref. year | Disaggregation |
| --- | --- | --- | --- | --- |
| Crop portfolio (% over total surface) | x_i_ | DRBA, (2016) and ITACyL, (2019) | 2015 | AWDU |
| Water applied (m^3^/ha) and irrigation technology | $w_{i}$ | DRBA, (2016) | 2015 | AWDU |
| Crop yield (kg/ha) | **y**_i_ | Adapted* from MAGRAMA (2015) | 2004 – 2015 | Agricultural District (*Comarca*) |
| Water availability (m^3^/ha) | W_g_ | DRBA, (2016a) | 1981 – 2018 | AWDU |
| Prices (EUR/kg) | p_i_ | MAGRAMA (2015) | 2004 – 201**5** | National (NUTS1) |
| Variable costs (EUR/ha) and subsidies (EUR/ha) | c_i_, s_i_ | Adapted* from MAPA (2019) | 2004 – 2015 | Agricultural District (*Comarca*) |
| Number of working days (days/ha) | TL_i_ and H_i_ | MAPA (2019) | 2004 – 2015 | Region (NUTS2) |

* Adapted means that the data was downscaled from original data provider. Downscaling from regional to agricultural district (*comarca*) was performed following the Annex I of the production regionalization plan (BOE, 1998).

#### **AI.4. calibration results**

Calibration results for the economic agents using the PMAMP model are presented in sheet “Calibration results” of the excel file available at this link: https://zenodo.org/doi/10.5281/zenodo.12783524

z_1_-z_5_ are the parameters of the Cobb-Douglas objective function, and e_a_, e_d,_ and e_m_ are the attribute, portfolio, and average calibration residuals, respectively. z_1_ is a relevant attribute in all the AWDUs; while Risk (z_2_) Tital Labor (z_3_), Hired Labor (z_4_), and Ratio of direct costs to total income (z_5_) are relevant only in some AWDUs.

Calibration residuals are considered low when the average calibration residual (e_m_) is below 10%, medium when it ranges between 10% and 20%, and high otherwise (Essenfelder et al., 2018; Parrado et al., 2019). Calibration residuals are low for all AWDUs but three that show medium residuals.

#### **AI.5. database**

The database used to calibrate the PMAMP model is in sheet “Database” of the excel file available at this link: https://zenodo.org/doi/10.5281/zenodo.12783524

## **Annex II: Macroeconomic model's supply, demand and data sources**

In the following model description, the time index has been omitted in the supply and demand side descriptions for the sake of readability since they relate to the behavior of the static model, while the time dimension is included in the recursive dynamic section.

#### **A.II.1 Supply side**

The supply side is modeled considering a representative firm for each sector. Each representative firm minimizes output costs (y) following a Leontief production function with two inputs: value added (va) and intermediate inputs (in):

| $\min_{va_{j,s},in_{j,s}} (pva_{j,s} va_{j,s}+pin_{j,s} in_{j,s})$ | (A.II.1) |
| --- | --- |

| $s.t.:y_{j,s}=min\left\{ va_{j,s}, in_{j,s} \right\}$ | (A.II.2) |
| --- | --- |

Where $pva_{j,s}$ is the price of the value added composite and $pin_{j,s}$ is the price of intermediate inputs in sector j of region s.

Value added is represented with a Constant Elasticity of Substitution (CES) function considering four primary factors (labor, capital, land and natural resources). Labor and capital are perfectly mobile within each region and used by all sectors. Land is a sector-specific production factor used only by agricultural sectors while natural resources, in a similar way, are used only by the extractive sector (extraction, fishing and forestry). Therefore, value added is a function of primary factors ($v_{f,j,s}$, with f = land, labor, capital, and natural resources), and the way these factors are combined is governed by a sector-specific substitution elasticity ($\sigma_{j}$). The parameter $\eta_{f,j,s}$ represents input augmenting (biased) technical change for each primary factor f in sector j and region s.

| $va_{j,s}=F\left( \eta_{f,j,s},v_{f,j,s}, \sigma_{j} \right) ; \sigma_{j}>0$ | (A.II.3) |
| --- | --- |

All primary factors are non-tradable commodities which implies that they are used domestically. While land allocation in Parrado et al. (2019) is modeled using a Constant Elasticity of Transformation (CET) function which allocates total available land in one region to all agricultural sectors according to sector-specific land rents, in this study changes in land allocation are set as exogenous for each agricultural sector, which allows using directly the land allocation determined by the microeconomic module.

#### **A.II.2 Demand side**

Each regional representative household earns income from primary factors and disposes it among private consumption (*Cons_s_*), government consumption (*Gov_s_*) and savings (*Sav_s_*) following a Cobb-Douglas per capita utility function (Hertel, 1997):

| $U_{s}=Cons_{s}^{\omega_{Cons}}{Gov}_{s}^{\omega_{Gov}}{Sav}_{s}^{\omega_{Ssv}}$ | (A.II.4) |
| --- | --- |

where *U_s_* represents utility in region s and parameters ω are the corresponding budget shares.

All regional savings are collected by a global bank which allocates them as investments among all regions of the world based on differences in regional rates of return. Investments are internationally mobile while the difference between savings and investments at the regional level determines the trade balance.

Trade flows are modeled in the domestic, intra-national and inter-national markets. For this purpose, the model considers an upper-level composite between domestic and imported goods and a lower-level composite that combines imports from all source regions. Following the GTAP model, this formulation assumes imperfect substitution ([Armington, 1969](https://www.sciencedirect.com/science/article/pii/S0048969720340481?via%3Dihub#bb0010)) between the domestic demand (ddj, s) and the aggregate demand for imported products (dm_j,s_) in region s and sector j via a CES function:

| $\min_{{dd}_{j,s},{dm}_{j,s}} ({pdd}_{j,s} {dd}_{j,s}+p{dm}_{j,s} {dm}_{j,s})$ | (A.II.5) |
| --- | --- |
| $s.t.: {dtot}_{j,s}=G\left( {dd}_{j,s},{dm}_{j,s}, \sigma_{j}^{Up} \right) ; \sigma_{j}^{Up}>0$ | (A.II.6) |

where *dtot_j,s_* is the total demand and *pdd_j,s_* and *pdm_j,s_* are the prices associated with domestic and aggregate demand for imported goods, respectively.

In order to add intra-national trade for the regions at the sub-country detail, the model includes aggregate imports (*dm_j,s_*) is which are sourced from the sub-country regions by means of a Constant Ratio of Elasticities of Substitution and Homothetic (CRESH) constraint (Cai and Arora, 2015; Hanoch, 1971; Pant, 2007) that provides flexibility in the choice of substitution when it comes to consider imports between a pair of spatial units.

| $\min_{{imp}_{j,s',s}} \sum_{s} pimp_{j,s',s} imp_{j,s',s}$ | (A.II.7) |
| --- | --- |
| $s.t.: {dm}_{j,s}=G_{2}\left( {imp}_{j,s}, \sigma_{j,s}^{Lo} \right) ; {imp}_{j,s}\in R^{S}, \sigma_{j,s}^{Lo}\in R^{S}, \sigma_{j,s',s}^{Lo}>0$ | (A.II.8) |

where *imp_j,s',s_* is the bilateral trade flow from region/country s′ to region/country s in sector j, *pimp_j,s',s_* is the associated import price; *imp_j,s_* are bilateral imports and *σ_j,s_^Lo^* are substitution elasticities of region/country s in sector j. with S being the number of countries/regions.

#### **A.II.3 Recursive dynamics: Capital accumulation**

The model generates a sequence of static equilibria under myopic expectations linked by capital accumulation between time periods. Growth is driven by changes in primary resources (capital, labor, land and natural resources). Dynamics are endogenous for capital and exogenous for other primary factors. Capital accumulation is the outcome of the interaction of investment allocation between regions. Savings are pooled by a world bank and allocated as regional investments according to:

| $\frac{{Inv}_{r,t}}{Y_{r,t}}=\omega_{r}exp\left[ \rho_{r}\left( r_{r,t}-r_{w,t} \right) \right]$ | (A.II.9) |
| --- | --- |

where: *Inv_r,t_* is regional annual investment, *Y_r,t_* is regional income, *r_r,t_* and *r_w,t_* are regional and world returns on capital respectively, *ω_r_* is a given parameter that represents the average propensity to save and *ρ_r_* is a flexibility parameter related to investment supply sensitivity to return differentials. The rationale of Eq. (A.II.9), follows the ABARE GTEM model (Pant et al., 2007). Capital stock accumulates over time in a standard relationship with a constant depreciation:

| $K_{r,t+1}=K_{r,t}-{\delta K}_{r,t}+{Inv}_{r,t}$ | (A.II.10) |
| --- | --- |

#### **A.II.4 Data sources**

The regionalized database has been developed by Bosello and Standardi, (2018), by extending the GTAP 8.1 database (Narayanan et al., 2012) , which is a collection of Social Accounting Matrices (SAMs) for 57 economic sectors and 134 countries (or groups of countries) in the world for the year 2007. The EU countries have been regionalized at NUTS 2 level and for this paper the regional aggregation shows 17 NUTS2 regions for Spain and 2 regions for the rest of the world (see Table 2 of the manuscript).

The original GTAP 8.1 database is available on the GTAP webpage as a free release at this [link](https://www.gtap.agecon.purdue.edu/databases/v8/).^[[1]](#footnote-1)^ The regionalized database is available upon request and maintained by the Centro Euro-Mediterraneo sui Cambiamenti Climatici. Additional information about the regionalized database is available at the ICES_REG model page at this [link](https://www.icesmodel.org/regionalized-model/).^[[2]](#footnote-2)^

## **Annex III: Results for rainfed and irrigated crops for each crop category j.**

Figure A.III.1 reports the results in terms of land use for each of the crop categories j of the macroeconomic model with a water charge increase of 0.1 EUR/m^3^ for both one-time and incremental adjustment. For the correspondence between crop categories j and individual crops, the reader can refer to Table 2 in the main text. It should be noted that the policy and baseline scenario share the same initial conditions in year 0 across all model setups—i.e., policy and baseline scenarios yield the same results. This initial condition is indicated across all figures with a black point. Note that since the “no coupling” and “static coupling” model setups are time-invariant, they jump from the initial condition in year 0 to the policy scenario simulation also in year 0, and results remain constant afterwards. Be aware that the scale changes between the different crops’ groups, and the graph for rainfed Sugar cane & beet is not reported as this crop is not cultivated as rainfed the study area.

| Irrigated Wheat | Rainfed Wheat |
| --- | --- |
| 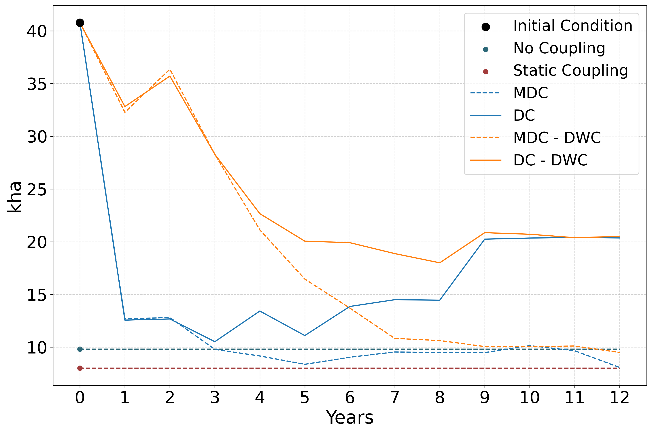 | 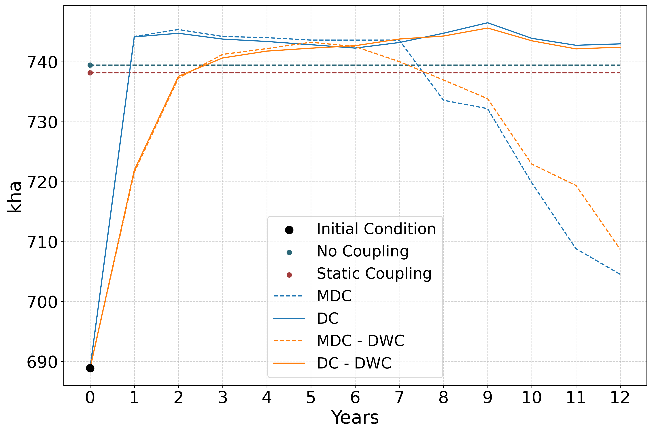 |
| Irrigated Other Cereals | Rainfed Other Cereals |
| 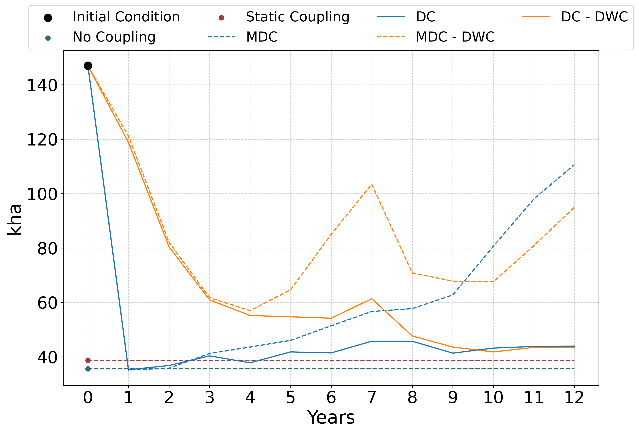 | 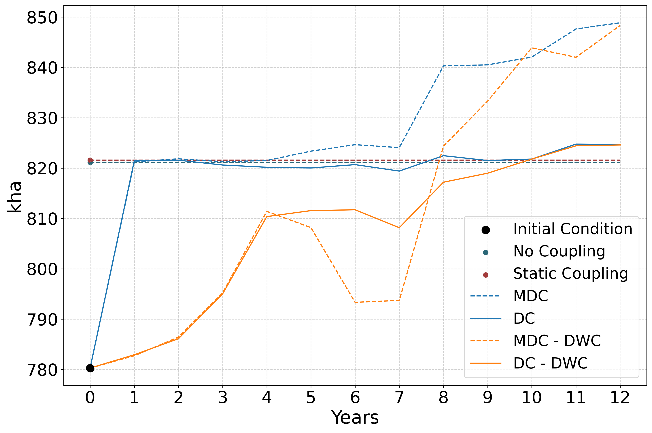 |
| Irrigated Vegetables & Fruits | Rainfed Vegetables & Fruits |
| 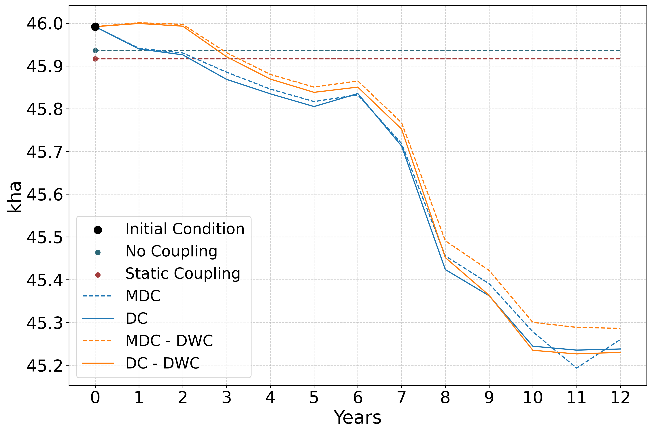 | 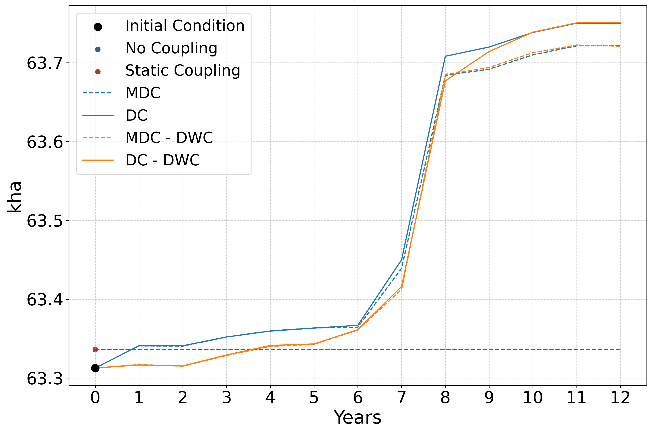 |
| Irrigated Oilseeds | Rainfed Oilseeds |
| 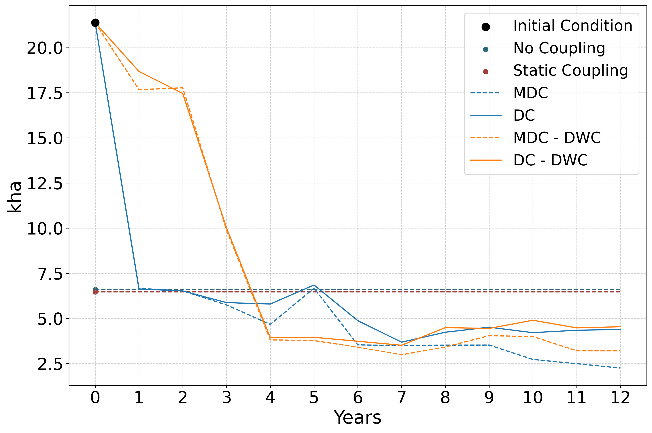 | 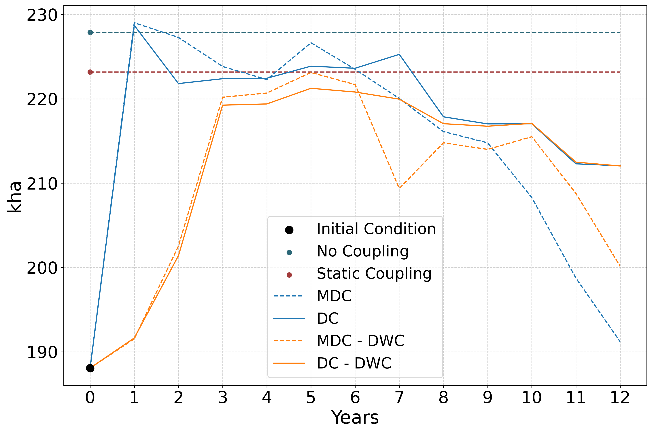 |
| Irrigated Sugar cane & beet | Rainfed Sugar cane & beet |
| 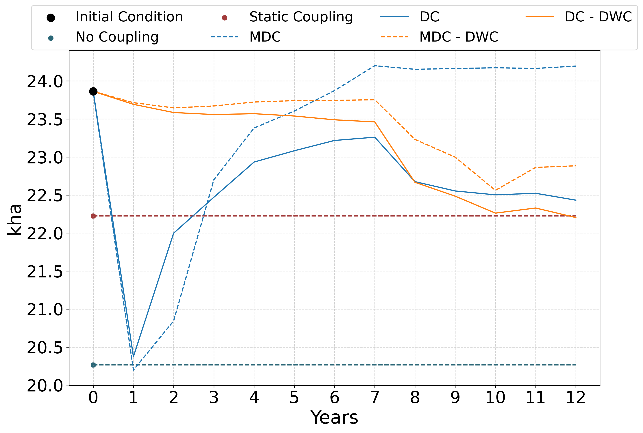 | N/A |
| Irrigated Crops not elsewhere classified | Rainfed Crops not elsewhere classified |
| 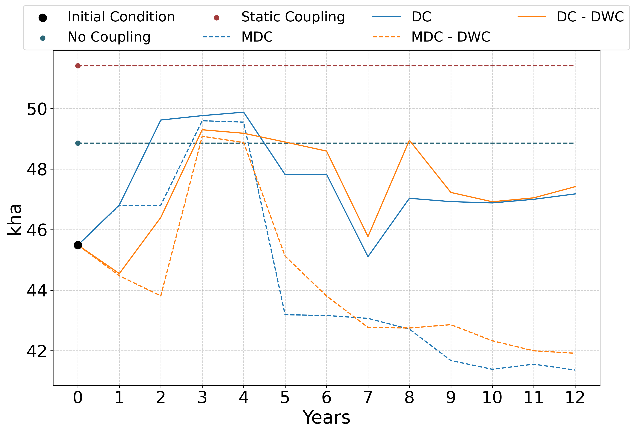 | 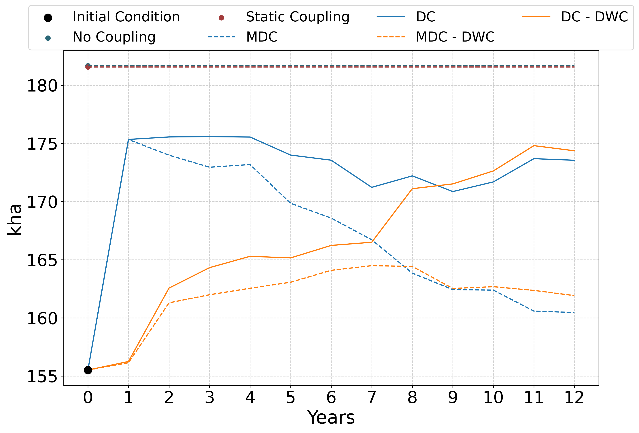 |

*Figure A.III.1. Land use changes for irrigated and rainfed crop category following a water charge increase of 0.1 EUR/m3.*

## **Literature**

Amador, F., Sumpsi, J.M., Romero, C., 1998. A non-interactive methodology to assess farmers’ utility functions: An application to large farms in Andalusia, Spain. Eur. Rev. Agric. Econ. 25, 92–102. https://doi.org/10.1093/erae/25.1.92

André, F.J., Herrero, I., Riesgo, L., 2010. A modified DEA model to estimate the importance of objectives with an application to agricultural economics. Omega, Empirical Research in the EU Banking Sector and the Financial Crisis 38, 371–382. https://doi.org/10.1016/j.omega.2009.10.002

André, F.J., Riesgo, L., 2007. A non-interactive elicitation method for non-linear multiattribute utility functions: Theory and application to agricultural economics. Eur. J. Oper. Res. 181, 793–807. https://doi.org/10.1016/j.ejor.2006.06.020

BOE, 1998. ORDEN de 21 de abril de 1998 relativa al Plan de Regionalización Productiva de España aplicable al sistema de pagos compensatorios a los productores de determinados cultivos herbáceos en la campaña (1998/1999).

Bosello, F., Standardi, G., 2018. A Sub-national CGE Model for the European Mediterranean Countries, in: Perali, F., Scandizzo, P.L. (Eds.), The New Generation of Computable General Equilibrium Models: Modeling the Economy. Springer International Publishing, Cham, pp. 279–308. https://doi.org/10.1007/978-3-319-58533-8_11

Cai, Y., Arora, V., 2015. Disaggregating electricity generation technologies in CGE models: A revised technology bundle approach with an application to the U.S. Clean Power Plan. Appl. Energy 154, 543–555. https://doi.org/10.1016/j.apenergy.2015.05.041

DRBA, 2016. Plan Hidrológico de la Cuenca del Duero 2015-2021 (River Basin Management Plan). Duero River Basin Authority, Valladolid (Spain).

Essenfelder, A.H., Pérez‐Blanco, C.D., Mayer, A.S., 2018. Rationalizing Systems Analysis for the Evaluation of Adaptation Strategies in Complex Human-Water Systems. Earths Future 6, 1181–1206. https://doi.org/10.1029/2018EF000826

Gómez-Limón, J.A., Gutiérrez-Martín, C., Riesgo, L., 2016. Modeling at farm level: Positive Multi-Attribute Utility Programming. Omega 65, 17–27. https://doi.org/10.1016/j.omega.2015.12.004

Gutiérrez-Martín, C., Gómez, C.M., 2011. Assessing irrigation efficiency improvements by using a preference revelation model. Span. J. Agric. Res. 9, 1009–1020. https://doi.org/10.5424/sjar/20110904-514-10

Hanoch, G., 1971. CRESH Production Functions. Econometrica 39, 695–712. https://doi.org/10.2307/1909573

Hertel, T.W. (Ed.), 1997. Global Trade Analysis: Modeling and Applications. Cambridge University Press, Cambridge; New York.

Inada, K.-I., 1963. On a Two-sector Model of Economic Growth: Comments and Generalization. Rev. Econ. Stud. 30, 119–127.

ITACyL, 2022. Mapa de Cultivos y Superficies Naturales de Castilla y León [WWW Document]. Inst. Tecnológico Agrar. Castilla Ón. URL http://mcsncyl.itacyl.es/es/inicio

Läpple, D., Kelley, H., 2013. Understanding the uptake of organic farming: Accounting for heterogeneities among Irish farmers. Ecol. Econ., Transaction Costs and Environmental Policy 88, 11–19. https://doi.org/10.1016/j.ecolecon.2012.12.025

MAGRAMA, 2022. Anuario de Estadística Agraria (Agricultural Statistics Yearbook) (Report). Ministerio de Agricultura, Alimentación y Medio Ambiente, Madrid (Spain).

MAPA, 2019. ECREA: Estudios de costes y rentas de las explotaciones agrarias (Report). Ministerio de Agricultura, Pesca y Alimentación, Madrid (Spain).

Marglin, S., 1967. Public Investment Criteria. Allen and Unwin and MIT Press, Studies in the Economic Development of India 4, London and Cambridge.

Narayanan, G.B., Aguiar, A., McDougall, R., 2012. Global Trade, Assistance, and Production: The GTAP 8 Data Base. Cent. Glob. Trade Anal. Purdue Univ.

Pant, H., 2007. GTEM: Global Trade and Environment Model (ABARE Technical Report). Australian Bureau of Agricultural and Resource Economics and Sciences, Canberra (Australia).

Parrado, R., Pérez-Blanco, C.D., Gutiérrez-Martín, C., Standardi, G., 2019. Micro-macro feedback links of agricultural water management: Insights from a coupled iterative positive Multi-Attribute Utility Programming and Computable General Equilibrium model in a Mediterranean basin. J. Hydrol. 569, 291–309. https://doi.org/10.1016/j.jhydrol.2018.12.009

Poppenborg, P., Koellner, T., 2013. Do attitudes toward ecosystem services determine agricultural land use practices? An analysis of farmers’ decision-making in a South Korean watershed. Land Use Policy, Themed Issue 1-Guest Editor Romy GreinerThemed Issue 2- Guest Editor Davide Viaggi 31, 422–429. https://doi.org/10.1016/j.landusepol.2012.08.007

Sampson, S.E., 1999. Axiomatic Justification for a Geometric Quality Aggregation Function. Decis. Sci. 30, 415–440. https://doi.org/10.1111/j.1540-5915.1999.tb01616.x

Sumpsi, J., Amador, F., Romero, C., 1997. On farmers’ objectives: A multi-criteria approach. Eur. J. Oper. Res. 96, 64–71. https://doi.org/10.1016/0377-2217(95)00338-X

1. https://www.gtap.agecon.purdue.edu/databases/v8/ [↑](#footnote-ref-1)
2. https://www.icesmodel.org/regionalized-model/ [↑](#footnote-ref-2)
